# Enhanced resistance to *Botryosphaeria dothidea* through upregulation of the lignin biosynthesis regulator *WRKY11* in poplar

**DOI:** 10.3389/fpls.2026.1737207

**Published:** 2026-02-26

**Authors:** Dongchen Shen, Jian Diao, Hui Lin, Wenjing Zhou, Wei Ma, Airong Dong, Ling Ma

**Affiliations:** 1 Forest Protection, College of Forestry, Northeast Forestry University, Harbin, China; 2 College of Wildlife and Protected Area, Northeast Forestry University, Harbin, China; 3 Pharmacy of Heilongjiang University of Chinese Medicine, Harbin, China

**Keywords:** *Botryosphaeria dothidea*, gene over-expression, lignin, poplar, WRKY transcription factor

## Abstract

The outbreak of poplar canker caused by *Botryosphaeria dothidea* poses a severe threat to poplar growth. Its resistance mechanisms are closely linked to the regulation of plant secondary metabolism and transcription factor-mediated defense pathways. However, as a plant-specific regulatory factor family, the functional mechanisms of the WRKY transcription factor (TF) family in poplar resistance to *B. dothidea* remain unclear. This study systematically elucidated the evolutionary characteristics of the WRKY gene family in *Populus trichocarpa* and their roles in disease resistance regulation in *P. davidiana* × *P. alba* var. *Pyramidalis* (Pdpap) through integrated genome-wide identification and molecular functional validation. Using BLASTp and Hidden Markov Model screening, 102 *PtrWRKYs* were identified. Phylogenetic analysis classified them into seven subfamilies based on *Arabidopsis thaliana* classification criteria. Functional diversification of this family was driven by plasticity in motif combinations, segmental duplication events, and subfamily-specific cis-regulatory elements. *PdpapWRKY11*, selected via RNA-seq and gene family analysis, significantly enhanced resistance to *B. dothidea* in transgenic Pdpap lines. Using the Pdpap–*B. dothidea* interaction system as a model, we further propose that *PdpapWRKY11* may activate key phenylpropanoid pathway genes (*PdpapPAL* and *PdpapCAD*), promoting lignin accumulation and thereby enhancing pathogen resistance. This research provides foundational insights into WRKY TF functions in poplar and establishes a theoretical basis for improving disease resistance for controlling canker disease.

## 1 Introduction

*Populus* (poplar), as one of the most important broad-leaved plantation species globally, plays a pivotal role in carbon sequestration forestry, ecological restoration, and biomass energy development due to its rapid growth, broad adaptability, and diversified economic value (Monson et al., 2020). Throughout its growth cycle, *Populus* faces persistent environmental stresses. Abiotic stresses including extreme temperatures, saline-alkaline conditions, and heavy metal pollution disrupt cellular physiology, inhibiting plant development (Chai and Schachtman, 2022; Waadt et al., 2022). Regarding biotic stresses, pathogen infection (fungi/bacteria) and insect herbivory impair fundamental processes like photosynthesis and nutrient translocation, ultimately causing substantial biomass loss (Tao et al., 2025; Qiu et al., 2025).

Among biotic stresses, Poplar canker, one of the most devastating stem diseases in global forestry, is primarily caused by fungal pathogens (Shi et al., 2025). Blister canker induced by *Botryosphaeria dothidea* (Ascomycota) poses the most severe threat (Liu et al., 2024). Notably, with the rapid expansion of intensive poplar plantations in recent years, the disease has exhibited exponential spread, leading to infection rates exceeding 95% and mortality rates surpassing 70% in heavily affected areas, thereby severely disrupting ecosystem functions (Ruess et al., 2021). It has now become a critical bottleneck constraining the sustainable development of the poplar industry. Pathological studies demonstrate that this host-dominant disease exhibits severity strongly correlated with host physiological status (lignin deposition capacity, endogenous hormone balance), immune response efficiency [expression levels of pathogenesis-related (PR) proteins], and environmental stress (drought-induced bark fissures). Characterized by prolonged latency, high recurrence rates, strong transmission capacity, and severe damage, it presents extreme challenges for containment (Xing et al., 2020).

To cope with environmental stresses, poplar has evolved multi-layered signaling networks involving coordinated mechanisms such as phytohormone signaling, reactive oxygen species (ROS) homeostasis, transcription factor (TF) cascades, and spatiotemporal regulation of secondary metabolites (Mertens et al., 2021; Sun et al., 2024). In studies on poplar stress resistance, the relevance of these coordinated mechanisms has been verified by multiple lines of evidence. For example, *Populus tomentosa* activates the jasmonic acid (JA) signaling pathway through the *PtoERF15*-*PtoMYC2b* transcriptional cascade to enhance its drought tolerance (Kong et al., 2023); the *PtoMYB99*-overexpressing lines accumulate higher levels of ROS under osmotic stress, and reduce poplar’s tolerance to osmotic stress by inhibiting the biosynthesis of abscisic acid (ABA) and JA (Long et al., 2024); *MYB134* coordinates the regulation of secondary metabolites to cope with both biotic and abiotic stresses by activating the proanthocyanidin (PAs) biosynthetic pathway (Mellway et al., 2009). In plant signaling networks, secondary metabolites are the core defensive substances for plants to resist environmental stresses. For instance, glucosinolates serve as key defensive metabolites in *Arabidopsis thaliana* against herbivorous insects and pathogen infections, while phenylpropanoids and flavonoids play important roles in plant defense against abiotic stresses. The synthesis dynamics of secondary metabolites directly determine the stress defense efficacy of the host plants (Carfora et al., 2025). These compounds are categorized into four major classes: phenolic compounds (lignin and flavonoids), terpenoids (monoterpenes and sesquiterpenes), nitrogen-containing metabolites (alkaloids), and sulfur-containing metabolites (glucosinolates) (Jahan et al., 2025). Within phenolic compounds, lignin rapidly deposits to form structural barriers (e.g., cell wall lignification), effectively blocking pathogen-derived extracellular enzymes (e.g., *UvPr1a* secreted by *Ustilaginoidea virens* in rice blast pathogenesis) and toxin diffusion (Chen et al., 2022). In contrast, flavonoids act as phytoalexins (e.g., genistein, daidzein, and formononetin that significantly inhibit *Diaporthe longicolla* virulence) to directly neutralize pathogens through hypersensitive responses (Zhuang et al., 2023; Qiu et al., 2024). The biosynthesis of both lignin and flavonoids originates from the shikimate pathway, which generates aromatic amino acid precursors (phenylalanine and tyrosine) via chorismic acid. These precursors are subsequently channeled into the phenylpropanoid pathway, bifurcating into two defensive branches that synergistically combat pathogen invasion (Wu et al., 2025).

Under biotic stress, the spatiotemporal accumulation of secondary metabolites is precisely regulated by TFs (Kajla et al., 2023). To date, at least 58 TF families have been identified in plants, among which six families (WRKY, MYB, bHLH, bZIP, AP2/ERF, and NAC) constitute the core regulatory module for stress responses. These TFs orchestrate secondary metabolite biosynthesis, transport, and storage by activating/repressing target genes. For instance, The *GhWRKY30*/*GhWRKY41*-*GhLac1* transcriptional cascade regulates lignin metabolism to enhance defense responses in *Gossypium hirsutum* (cotton) (Xiao et al., 2024). Hormonal (JA/SA) and ROS signaling pathways coordinate the balance between defense metabolism and growth. A representative example includes wound-induced JA in *A. thaliana*, which activates ABA biosynthesis to promote lignin deposition (Xu HM, et al., 2024), while *Pseudomonas syringae* infection in soybean triggers BiP overexpression, upregulates *GmNAC32* to mediate SA-dependent flavonoid accumulation (Rodrigues et al., 2021). Emerging evidence highlights the central role of WRKY TFs in differentially regulating lignin and flavonoid biosynthesis via phenylpropanoid pathway genes across species. Key examples include: *CcWRKY25* from *Capsicum chinense* (pepper), whose heterologous expression in *A. thaliana* enhances the expression of phenylpropanoid genes (*AtPAL*, *At4CL*), thereby elevating lignin and flavonoid levels (Zhang et al., 2023). Group IIc WRKYs in *Gossypium hirsutum*, which induce the *GhMKK2*-*GhNTF6* pathway to boost flavonoid biosynthesis against *Fusarium oxysporum* infection (Wang LJ, et al., 2022). *NtWRKY28* in *Nicotiana tabacum* (tobacco), whose overexpression activates phenylpropanoid genes to accumulate lignin and flavonoids in response to green peach aphid feeding (Chu et al., 2025). Defined by their conserved N-terminal WRKYGQK motif and C-terminal zinc finger domains (Xiao et al., 2023), WRKY TFs represent a plant-specific regulatory protein family. Deciphering these complex regulatory networks will advance our theoretical understanding of plant stress adaptation and provide a scientific foundation for precision breeding strategies leveraging gene editing technologies.

Although significant progress has been made in understanding the molecular mechanisms by which WRKY TFs regulate the biosynthesis of secondary metabolites to enhance plant immunity, research on the molecular regulatory network underlying poplar, in response to *B. dothidea* infection remains lagging compared to other plant species. The central regulatory hubs governing canker resistance, including WRKY-mediated transcriptional regulation, ROS homeostasis, and the coordinated mechanisms of lignin/flavonoid biosynthesis pathways, have yet to be systematically elucidated, hindering the precision design of disease-resistant breeding strategies (Xu et al., 2025). We hypothesized that WRKY TFs in poplar coordinate lignin and flavonoid biosynthesis to enhance resistance against *B. dothidea*. To test this hypothesis, we systematically analyzed 102 WRKY TFs (PtrWRKYs) in *Populus*, including promoter cis-acting element prediction, chromosomal localization, segmental duplication event identification, and multi-species collinearity analysis. We identified a gene encoding the WRKY family protein *PdpapWRKY11*, which was significantly upregulated upon *B. dothidea* induction. Phenotypic and physiological analyses demonstrated that overexpression of *PdpapWRKY11* in Pdpap enhanced its resistance to *B. dothidea*. We also measured key enzymes and biosynthetic genes governing the lignin and flavonoid metabolic pathways, thereby proposing a regulatory network through which *PdpapWRKY11* mediates poplar defense against *B. dothidea*. This study provides a foundational framework for future research on these 102 WRKY TFs and offers insights for improving stress resistance in poplar.

## 2 Materials and methods

### 2.1 Genome-wide identification of *PtrWRKYs*

The genome of *Populus trichocarpa* was retrieved from Phytozome v12.1. The amino acid sequences of *A. thaliana* WRKY proteins were downloaded from TAIR (https://www.arabidopsis.org/). Using these known *A. thaliana* WRKY proteins as query sequences, a BLASTP search (v2.2.28; E-value ≤ 1×e^-10^) was performed against the *P. trichocarpa* genome database. Concurrently, WRKY genes were identified using HMMER 3.0 with the WRKY domain (PF03106) Hidden Markov model (HMM, E-value ≤ 1×e^-10^) from the Pfam database (http://pfam.xfam.org). We took the intersection of the results from these two approaches. Sequences lacking intact WRKYGQK motifs or zinc finger domains (C_2_HC/C_2_H_2_ type) were filtered out via multiple sequence alignment and validated using SMART 9.0 (http://smart.embl-heidelberg.de/) and NCBI Conserved Domain Database (CDD).

### 2.2 Bioinformatics analysis of *PtrWRKYs*

The physicochemical properties and subcellular localization of *P. trichocarpa* WRKY proteins were predicted using ProtParam (https://web.expasy.org/protparam/) and WoLF PSORT (https://wolfpsort.hgc.jp/). The amino acid sequences of WRKY genes from *P. trichocarpa* and *A. thaliana* were selected for phylogenetic relationship analysis. A phylogenetic tree was constructed using the Maximum Likelihood (ML) method in MEGA12 software (with 1000 bootstrap replicates), and optimization was conducted using the iTOL platform (https://itol.embl.de/). Conserved motifs were identified using MEME (http://meme-suite.org/), with the maximum number of motifs set to 10, the minimum motif width to 6, and the maximum motif width to 50. Functional annotation was performed with InterProScan (https://www.ebi.ac.uk/interpro/). Secondary structures were predicted using SOPMA (https://npsa-prabi.ibcp.fr/cgi-bin/npsa_automat.pl?page=npsa_sopma.html), tertiary structures were generated using SWISS-MODEL (https://swissmodel.expasy.org/), and topological heterogeneity models were analyzed using Protter (http://wlab.ethz.ch/protter/start/). Tandem and segmental duplication events within the *P. trichocarpa* WRKY gene family were analyzed using MCScanX (Python) with default parameters (BLASTP E-value< 1×e^-10^, match score >50). Chromosomal localization of WRKY genes was visualized using TBtools v2.210. Collinearity relationships between *P. trichocarpa* and *A. thaliana*, *Solanum lycopersicum* (GCF_000188115.5), *Eucalyptus grandis* (GCF_016545825.1), and *Glycine max* were performed using the Dual Synteny Plotter (TBtools v2.210). Promoter regions of WRKY genes (2000 bp upstream of the start codon) were extracted. Cis-regulatory elements within these promoter regions were predicted using the PlantCARE database (https://bioinformatics.psb.ugent.be/webtools/plantcare/html/). Ka/Ks ratios for duplicated gene pairs were calculated using KaKs_Calculator (v2.0). Genomic data for all species were obtained from Phytozome v12.1 (https://phytozome-next.jgi.doe.gov/) and the NCBI (https://www.ncbi.nlm.nih.gov/).

### 2.3 Plant materials and growth conditions

Wild-type (WT) *Populus davidiana* × *P. alba* var. *Pyramidalis* (Pdpap) provided by the Department of Forest Protection, Northeast Forestry University (Harbin, Heilongjiang, China) was used for genetic transformation. Leaves from 1- to 2-month-old Pdpap seedlings were placed on differentiation medium (0.5 g·L^-1^ 6-BA; 0.1 g·L^-1^ NAA; 2.47 g·L^-1^ 1/2MS basal medium; 20 g·L^-1^ sucrose; pH 6.0) and cultured for 2 months, then transferred to rooting medium (2.47 g·L^-1^ 1/2MS basal medium; 0.01 mg·L^-1^ NAA; 20 g·L^-1^ sucrose; pH 6.0). After an additional 2 months, plants were acclimatized in a soil culture room. Both tissue culture and soil culture rooms were maintained at 22°C with a 16/8 h (light/dark) photoperiod and 70% relative humidity. Soil-cultured seedlings of Pdpap with consistent growth status were selected for subsequent treatments.

### 2.4 Isolation, culture and Inoculation of *B. dothidea*

*B. dothidea* was isolated from canker-infected poplar stems collected at the demonstration forestry base of Northeast Forestry University. Pathogen purification was performed using tissue isolation on PDA medium (200 g·L^-1^ potato infusion; 15 g·L^-1^ agar; 20 g·L^-1^ glucose; pH 7.0). The strain was deposited in the China General Microbiological Culture Collection Center (CGMCC 3.27823). *B. dothidea* was cultured on PDA for 7 d until sporulation. For inoculation, a 6-mm epidermal incision was made on the stem of Pdpap seedlings 2–3 cm above the soil surface. A 6-mm mycelial plug of *B. dothidea* was placed on the incision, while a sterile PDA plug served as the control. Growth conditions for Pdpap were identical to those described in Section 2.3. Leaf samples were collected at 0, 6, 12, 24, and 48 h post-inoculation to analyze the expression pattern of *PdpapWRKY11* during infection. Three biological replicates were included for each time point. Additionally, root, stem, and leaf samples were collected at 0 h for spatial expression profiling, with three biological replicates per tissue.

### 2.5 cDNA cloning and sequence analysis of *PdpapWRKY11*

Total RNA was extracted from Pdpap using the RNAprep Pure Plant Plus Kit (Tiangen, Beijing, China), and cDNA synthesized with TransScript First-Strand cDNA Synthesis SuperMix (TransGen, Beijing). *PdpapWRKY11* was amplified with gene-specific primers under the following PCR conditions: initial denaturation at 95°C for 5 min; 35 cycles of 94°Cfor 30 s (denaturation), 58°C for 30 s (annealing), 72°C for 1 min (extension); followed by a final extension at 72°C for 10 min, gel-purified (EasyPure^®^ Quick Gel Extraction Kit), cloned into DH5α (Vazyme Biotech Co., Ltd., Nanjing, China), and sequenced (Primer sequences are listed in **Supplementary Table 1**). Evolutionary relationships were analyzed via BLAST on NCBI, and phylogenetic trees were built using MEGA X (neighbor-joining method). Amino acid sequence alignment was performed with DNAMAN 9.

### 2.6 Quantitative real-time PCR analysis

Total RNA was extracted from samples obtained in Section 2.4 using the method described in Section 2.5. Subsequently, cDNA was synthesized to analyze the expression pattern of *PdpapWRKY11* during *B dothidea* infection and its spatial expression profile. qPCR was performed using SYBR Green PCR Master Mix (Vazyme Biotech Co., Ltd., Nanjing, China) and gene-specific primers, with 100 ng of cDNA template per amplification reaction. Three independent biological replicates were analyzed per group, and *PdpapEF1-α* served as the reference gene for qPCR normalization. Relative expression levels were calculated using the 2^−ΔΔCT^ method (primer sequences are listed in **Supplementary Table 1**).

### 2.7 Subcellular localization assay

The fusion construct 35S::PdpapWRKY11-GFP (containing the *PdpapWRKY11* coding sequence and GFP) and control 35S-GFP were transiently transformed into *A. thaliana* protoplasts using established protocols. Localization was observed under a Nikon C2-ER confocal microscope (Nikon Instruments Inc., Tokyo, Japan) with excitation at 488 nm and emission detection at 510 nm.

### 2.8 Construction and validation of putative transgenic Pdpap lines

The *PdpapWRKY11* gene obtained in Section 2.5 was cloned into the pBI121 vector (*SpeI*/*XmaI* sites) under the 35S promoter. The recombinant plasmid (pBI121-*PdpapWRKY11*) was transformed into *Agrobacterium tumefaciens* EHA105 (Verdure Biotech Co., Ltd., Nanjing, China). Bacterial cultures (OD_600_ = 0.5–1.0) were used for leaf disc transformation of WT poplar. Transformed explants were cultured for 2 months followed by 1 month soil acclimatization under conditions described in Section 2.3. Genomic DNA was extracted from 3-month-old transformant lines using a Super Plant Genomic DNA DP360 Kit (Tiangen Biotech, Beijing) and subjected to PCR verification with gene-specific and vector primers (*PdpapWRKY11*-F*/*pBI121-R, *PdpapWRKY11*-R*/*pBI121-F). Controls included: recombinant plasmid (positive control), WT plants (negative control), and ddH_2_O (no-template control). The PCR program followed the protocol described in Section 2.5. qPCR was further performed to confirm transcription levels (primers in **Supplementary Table 1**).

### 2.9 Growth and physiological indices

To evaluate the impact of *PdpapWRKY11* overexpression on *B. dothidea* resistance, two-month-old WT and transformant lines (OE4, OE7, OE10) were inoculated with *B. dothidea* at three months of age using the protocol described in Section 2.4. Plants were maintained under growth conditions identical to Section 2.3 for 20 d. Fresh weight and root length were measured at 0, 5, 10, 15, and 20 d, with three biological replicates per group. Baseline values (0 d WT) were normalized to 1(relative value normalization). Concurrently, disease indices were statistically analyzed for both WT and transformant lines. Based on the disease progression observed in the plants, lesions were classified into five severity grades: Grade 0 (healthy/no lesions), Grade 1 (lesion width< 1/3 of stem circumference), Grade 2 (lesion width 1/3–1/2 of stem circumference), Grade 3 (lesion width 1/2–3/4 of stem circumference), and Grade 4 (lesion width > 3/4 of stem circumference or plant mortality). The disease index was calculated using the formula (Jilin Provincial Administration for Market Regulation (JQTS), 2013):


$$\begin{array}{c} \text{Disease Index }\left(\%\right)=[\Sigma \;\left(\text{Disease grade}\times \text{Number of plants per grade}\right)\\ /\left(\text{Total plants surveyed}\times \text{Maximum disease grade}\right)]\times 100 \end{array}$$

For each enzyme assay, 0.05 g of leaf tissue was pulverized in liquid nitrogen and homogenized in 0.1 mol/L phosphate buffer (pH=7.0). The homogenate was centrifuged at 10,000 × g for 10 min at 4 °C, and the supernatant was used for measurements. Peroxidase (POD) activity was quantified by colorimetric assay (Li et al., 2013), superoxide dismutase (SOD) by the hydroxylamine method (Zhao et al., 2013), malondialdehyde (MDA) content by the thiobarbituric acid (TBA) method (Liu et al., 2010), and hydrogen peroxide (H_2_O_2_) levels by colorimetric assay (Liu et al., 2013), using commercial kits from Nanjing Jiancheng Bioengineering Institute (Nanjing, China) strictly adhering to the manufacturer’s protocols. The corresponding catalog numbers were A084-3-1, A001-1-2, A003-3-1, and A064-1-1.

### 2.10 Lignin and flavonoid quantification

Leaf samples (0.05 g) were collected at 0, 6, 12, 24, and 48 h post-*B. dothidea* inoculation, pulverized in liquid nitrogen, and extracted with 2 mL of 60% ethanol under agitation at 60 °C for 2 h (Xu J, et al., 2021). After centrifugation at 10,000 × g (room temperature, 10 min), the supernatant was collected for flavonoid quantification using a colorimetric assay kit (Nanjing Jiancheng Bioengineering Institute, China; Cat. No. A142-1-1). Stem samples collected at 0, 5, 10, 15, and 20 d post-inoculation were oven-dried at 80 °C, pulverized, sieved through a 30-mesh filter, and 5 mg aliquots were used for lignin content determination via the acetyl bromide method (Solarbio Science & Technology Co., Ltd., Beijing, China; Cat. No. BC4200) with three biological replicates (Hu et al., 2022).

### 2.11 Lignin pathway enzyme activity and gene expression

Leaf samples (0.05 g) were collected at 0, 6, 12, 24, and 48 h post-*B. dothidea* inoculation for each enzyme assay. Phenylalanine ammonia-lyase (PAL) activity (Liu et al., 2005) was quantified using a colorimetric assay kit (Nanjing Jiancheng Bioengineering Institute, China; Cat. No. A137-1-1), while cinnamyl alcohol dehydrogenase (CAD) activity (De Ascensao et al., 2000) was determined via an NADH-dependent activity assay kit (Boxbio Science & Technology Co.,Ltd., Beijing, China; Cat. No. AKSU053M). RNA from these samples was used for qRT-PCR analysis of *PdpapPAL* and *PdpapCAD* expression, with *PdpapEF1-α* as reference genes (primer sequences listed in **Supplementary Table 1**).

### 2.12 Statistical analysis

The qRT-PCR and physiological data were analyzed using two-way ANOVA and one-way ANOVA (SPSS 24.0, IBM), followed by Student’ s t-test for pairwise comparisons, with a *P*-value< 0.05 considered statistically significant.

## 3 Results

### 3.1 Identification and characterization of the *PtrWRKYs* gene family in *P. trichocarpa*

**Figure 1:**
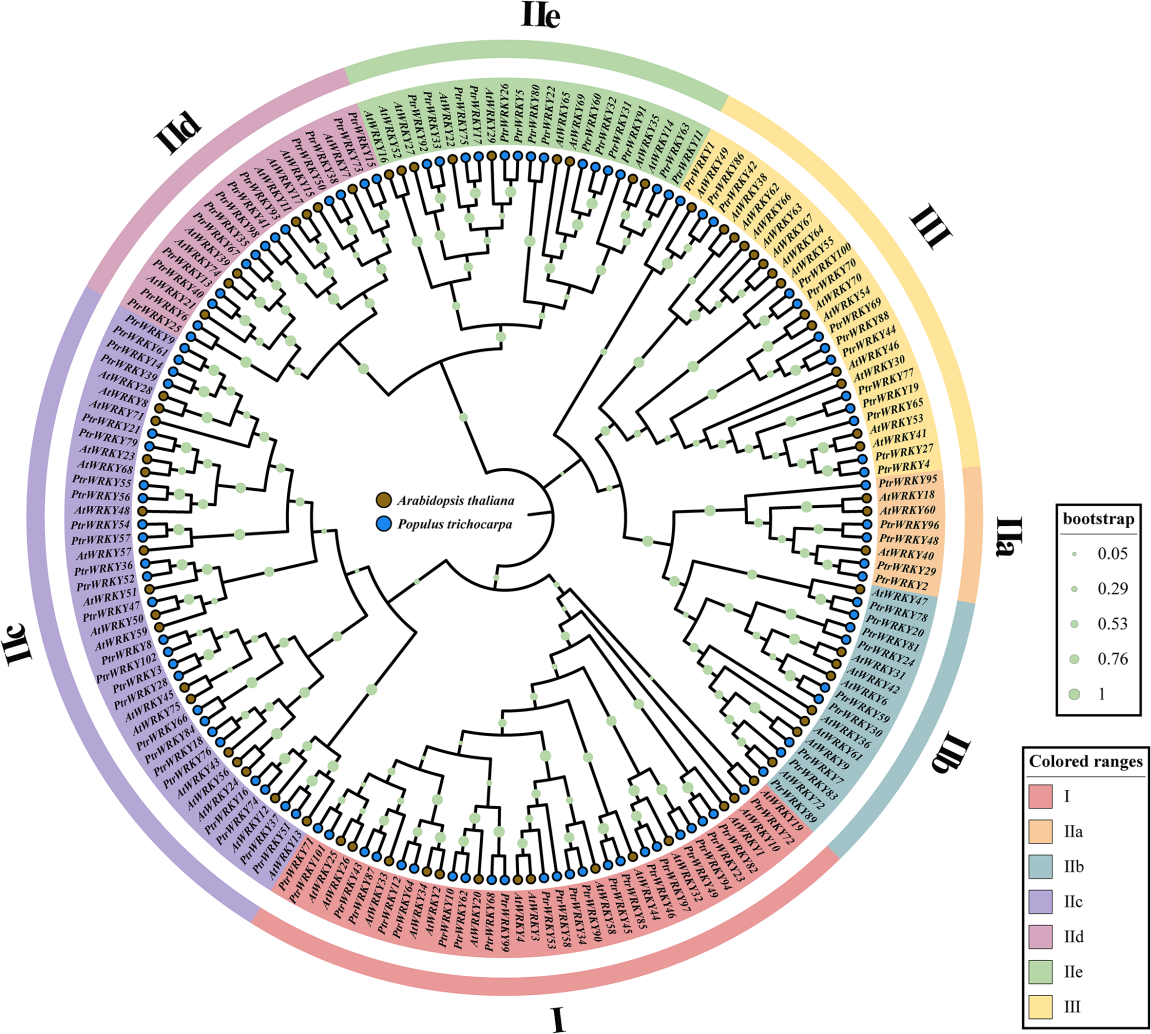
Phylogenetic analysis of WRKY TFs in *Populus trichocarpa* and *Arabidopsis thaliana*. A phylogenetic tree was constructed using the Maximum Likelihood (ML) method in MEGA 12 based on protein sequences of 72 *AtWRKYs* and 102 *PtrWRKYs*. Distinct clades are color-coded as follows: red (I), orange-brown (IIa), blue (IIb), purple (IIc), pink (IId), green (IIe), and yellow (III). Gene IDs are labeled to indicate species-specific distribution, with blue nodes representing *PtrWRKYs* and brown nodes representing *AtWRKYs*.

In *P. trichocarpa*, 102 WRKYs were systematically identified and designated as *PtrWRKYs* (numbered according to their genomic positions in the *P. trichocarpa* genome; **Supplementary Table 2**). To elucidate evolutionary relationships among *PtrWRKYs*, a ML phylogenetic tree was constructed using amino acid sequences from *P. trichocarpa* and *A. thaliana*, enabling subfamily classification (**Figure 1**). According to the phylogenetic topology and classification criteria for *A. thaliana* WRKYs, the *PtrWRKYs* were stratified into seven distinct clades, with each clade color-coded for visualization (Robatzek et al., 2000). Subfamily IIc exhibited the largest membership (25 *PtrWRKYs*), whereas subfamily IIa contained the fewest members (5 *PtrWRKYs*). Subfamilies I, IIb, IId, IIe, and III comprised 23, 9, 13, 14, and 13 *PtrWRKYs*, respectively. Notably, homologs of *AtWRKYs* were randomly distributed across all subfamilies.

The physicochemical properties of PtrWRKYs exhibited substantial variation across different subfamilies, while members within the same group demonstrated highly consistent characteristics (**Supplementary Table 3**). The PtrWRKY proteins had an average length of 371 amino acids (range: 135–732 aa) with corresponding molecular weights averaging 41.00 kDa (range: 15.54–78.91 kDa). Theoretical isoelectric points (pI) spanned from 5.08 to 9.86, and aliphatic indices varied between 31.85 and 82.00. Instability index analysis revealed values of 37.83–70.28, with 92% of proteins classified as unstable (>40) and only a minority as stable (<40). All PtrWRKY members exhibited hydrophilic properties, as evidenced by their grand average of hydropathicity (GRAVY) scores between -1.34 and -0.36.

To investigate the structural characteristics and functional evolution of the PtrWRKYs, this study revealed the co-evolutionary patterns between molecular architecture and functional modules through integrated analyses of Motif Pattern, Conserved domain, and Gene structure (**Supplementary Figure 1**). MEME (Multiple Expectation Maximization for Motif Elicitation) identified 10 conserved motifs (Motif1-Motif10) in the PtrWRKYs, annotated via Pfam. Motif1 contained the canonical WRKYGQK domain, while Motif2/4/6 formed the DNA-binding framework, with Motif4 harboring a zinc finger. Motif5 exhibited intrinsic disorder, Motif8 was classified into the PRK13922 super family, and the remaining motifs lacked functional annotations. Phylogenetic clustering revealed that each PtrWRKY gene carries 2–8 motifs, with evolutionarily related members displaying highly conserved motif combinations. The ubiquitous presence of Motif2 across all PtrWRKYs, subfamily-specific distributions (e.g., Motif5 enriched in subfamilies I/IIb/IIc, Motif8 predominant in IIa/IIb, Motif9 exclusive to IIa/IIb/IIe, and Motif10 unique to subfamily I).

Conserved Domain analysis further uncovered structural evolutionary signatures: while subfamily I retains dual WRKY domains, other subfamilies possess a single WRKY domain. Acquired domains, including Plant_zn_clust in IId (zinc binding), FlaE domain in WRKY89 (flavonoid synthesis), Nucleoporin_FG2 superfamily in WRKY100 (transport through nuclear pore), and ODAM superfamily (enamel formation) in WRKY30, indicate domain-mediated functional specialization. Gene structure analysis revealed conserved genomic architectures among subfamily members, with intron numbers ranging from 1 to 6 (predominantly 2-4) across different subgroups. The results demonstrated that subfamilies I, IIa, IIb, IIc, IId, IIe, and III contained 2-6, 2-4, 3-6, 1-3, 2-3, 1-3, and 2 introns, respectively. The findings collectively demonstrate that the functional diversity of the WRKY family arises from the synergistic interplay of motif combinatorial patterns, domain variations, and exon-intron structural divergence.

Secondary structure analysis (**Supplementary Table 4**) revealed that PtrWRKY family proteins predominantly consist of *α*-helices (13.78%, 49 amino acids on average), extended strands (6.72%, 24 amino acids), and random coils (79.50%, 295 amino acids), with no *β*-turns detected. Structural heterogeneity was observed among members: *PtrWRKY29* exhibited the highest α-helix proportion (29.47%), *PtrWRKY1* displayed the most extended strands (10.37%), and *PtrWRKY64* showed exceptionally high random coil content (87.79%), suggesting potential conformational flexibility. The tertiary structure modeling (**Supplementary Table 5**) was performed using high-similarity templates from the AlphaFold Database, with sequence identities ranging from 70.13% to 100.00% (average 89.80%). All models were validated as high-confidence structures.

The topological heterogeneity model analysis revealed that no transmembrane helices were identified in any PtrWRKY proteins (**Supplementary Table 6**). Specifically, 13 PtrWRKYs lacked N-glycosylation sites, whereas 89 PtrWRKYs contained 0–12 predicted N-glycosylation sites. None of the PtrWRKYs exhibited detectable signal peptide sequences. Subcellular localization predictions demonstrated that the majority of PtrWRKYs were nuclear-localized (**Supplementary Table 7**). Notably, *PtrWRKY99* was predicted to reside in the cytoplasm, *PtrWRKY18* and *PtrWRKY76* in peroxisomes, and *PtrWRKY90* in chloroplasts.

The chromosomal distribution of PtrWRKYs in poplar exhibits pronounced non-randomness and spatial heterogeneity (**Supplementary Figure 2**). These genes are distributed across 18 out of 19 chromosomes, with complete absence on Chr09, suggesting potential inhibitory epigenetic regulatory mechanisms suppressing WRKY gene evolution or the absence of adaptive evolutionary pressures on this chromosome. Chr01 and Chr02 display significant gene enrichment, each harboring 12 PtrWRKY members (accounting for 11.76% of the family). Notably, gene distribution density shows no correlation with chromosomal length: the shorter chromosome 6 (approximately 50.2 Mb) contains 9 genes, while the longer chromosome 16 (72.8 Mb) accommodates only 4 members. Remaining chromosomes demonstrate graded distribution patterns: chromosomes 5, 12, and 17 each carry 6 genes; chromosomes 3, 4, and 14 contain 5 each; chromosomes 7, 8, 10, and 19 host 3 each; chromosomes 13 and 16 possess 2 members.

**Figure 2:**
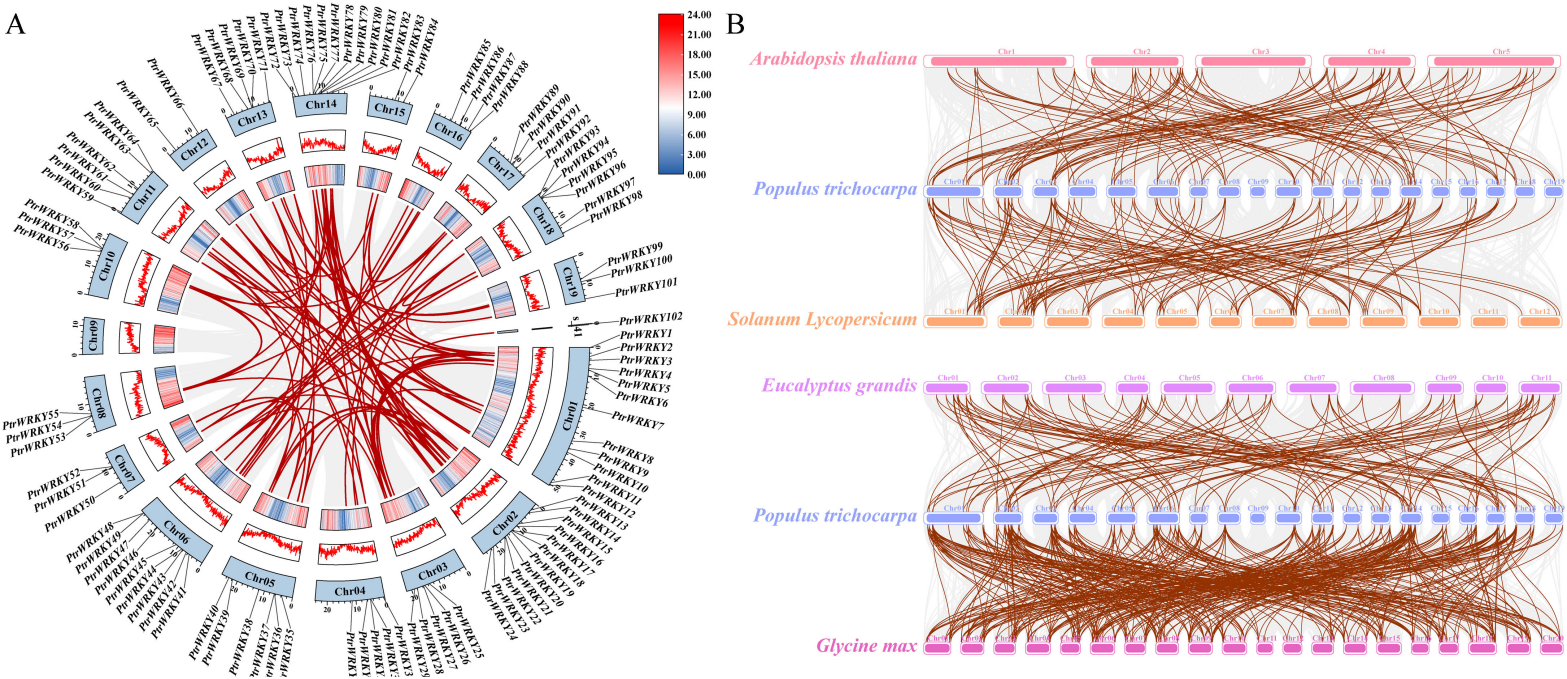
Syntenic analysis of PtrWRKYs. **(A)** Intrachromosomal syntenic relationships among PtrWRKYs in *Populus trichocarpa*. Chr1–Chr19 are labeled numerically. Red lines highlight collinear PtrWRKYs gene pairs, while gray lines represent all syntenic blocks within the *P. trichocarpa* genome. **(B)** Cross-species collinearity between PtrWRKYs and their orthologs in four other plant species. Brown lines denote syntenic pairs of PtrWRKYs and their orthologous counterparts, whereas gray lines indicate genome-wide orthologous synteny between P. trichocarpa and the compared species.

A substantial proportion of plant gene duplication arises from interchromosomal replication. To elucidate the evolutionary origins and expansion mechanisms of the PtrWRKYs in poplar, this study integrates intra- and inter-species collinearity analyses to reveal its duplication patterns. Intra-species collinearity analysis using MCScanX identified extensive segmental duplication events among PtrWRKYs, with 58 core genes forming 95 homologous duplication pairs (**Figure 2A**). These pairs, interconnected by red lines in the synteny map, are distributed across 18 chromosomes harboring PtrWRKYs and exhibit interchromosomal interaction features, indicating that interchromosomal recombination dominates gene family expansion.

Collinearity-based inference suggests that one gene in each duplicated pair originated from segmental duplication of the other. The Nonsynonymous to synonymous substitution ratio (Ka/Ks), a metric for evolutionary rate and selective pressure, revealed that all duplication pairs exhibit Ka/Ks values below 1 (mean = 0.24, range: 0.07–0.53), strongly supporting purifying selection.

To assess cross-species conservation of WRKY TFs, we constructed comparative synteny maps between *P. trichocarpa* and four representative dicot species (*A. thaliana*, *Solanum lycopersicum*, *Eucalyptus grandis*, and *Glycine max*) (**Figure 2B**). Results demonstrate broad evolutionary conservation between PtrWRKYs and their dicot homologs, with the highest collinearity observed in *G. max* (soybean, 400 homologous events), followed by *E. grandis* (149 events), *S. lycopersicum* (142 events), and *A. thaliana* (118 events). In summary, both interchromosomal segmental duplication events and functional conservation of the gene family influence the functional differentiation of genes in PtrWRKYs.

To better understand the biological processes involved in PtrWRKYs, this study systematically revealed subfamily-specific regulatory networks by analyzing 2,000 bp cis-acting elements in the promoter regions of PtrWRKYs (**Supplementary Figure 3**). A total of 27 functional cis-acting elements were identified, with each gene harboring 19–66 elements (mean = 34), which were classified into four core regulatory modules: (1) stress response module, including hypoxia/anaerobic, drought, pathogen, and pest stress elements; (2) growth and development module, such as meristem expression, endosperm expression, root-specific elements, and differentiation of palisade mesophyll cells; (3) hormone response module, encompassing ABA, methyl jasmonate (JA signaling), auxin, and salicylic acid (SA); (4) photoperiod regulation module containing light-responsive elements and circadian control. Notably, all PtrWRKYs carried these four element categories but exhibited significant functional divergence among subfamilies: in biotic stress response elements, subfamilies IIa/IIb/IIc were enriched with as-1 (biotic stress response), while IIe/IId preferentially contained WRE3 (wound response), and IId/III subfamilies specifically accumulated WUN-motif (damage response). Hormone signaling elements (MYC, ARE) were highly enriched in I/III/IIe subfamilies, whereas growth and development-related elements exhibited the lowest overall proportion. These findings demonstrate that the functional expression of the PtrWRKYs genes is regulated by cis-acting elements associated with light, phytohormones, stress, and plant development.

Based on phylogenetic and functional modular analyses of the WRKY gene family in *P. trichocarpa*, we found that multiple subfamilies (e.g., IId and III) were significantly enriched with stress-responsive cis-elements, suggesting their potential central roles in stress resistance regulation in the *Populus* genus.

To validate the biological functions of WRKY TFs in poplar-pathogen interactions, this study employed Pdpap as the experimental system. This hybrid poplar exhibits strong innate disease resistance against the canker pathogen *B. dothidea* and possesses well-established genetic transformation protocols. By integrating evolutionary theoretical findings from *P. trichocarpa* with functional validation in this high-resistance hybrid system, this study advanced the elucidation of plant disease resistance mechanisms.

### 3.2 Overexpression of *PdpapWRKY11* enhances resistance against *B. dothidea* in Pdpap

**Figure 3:**
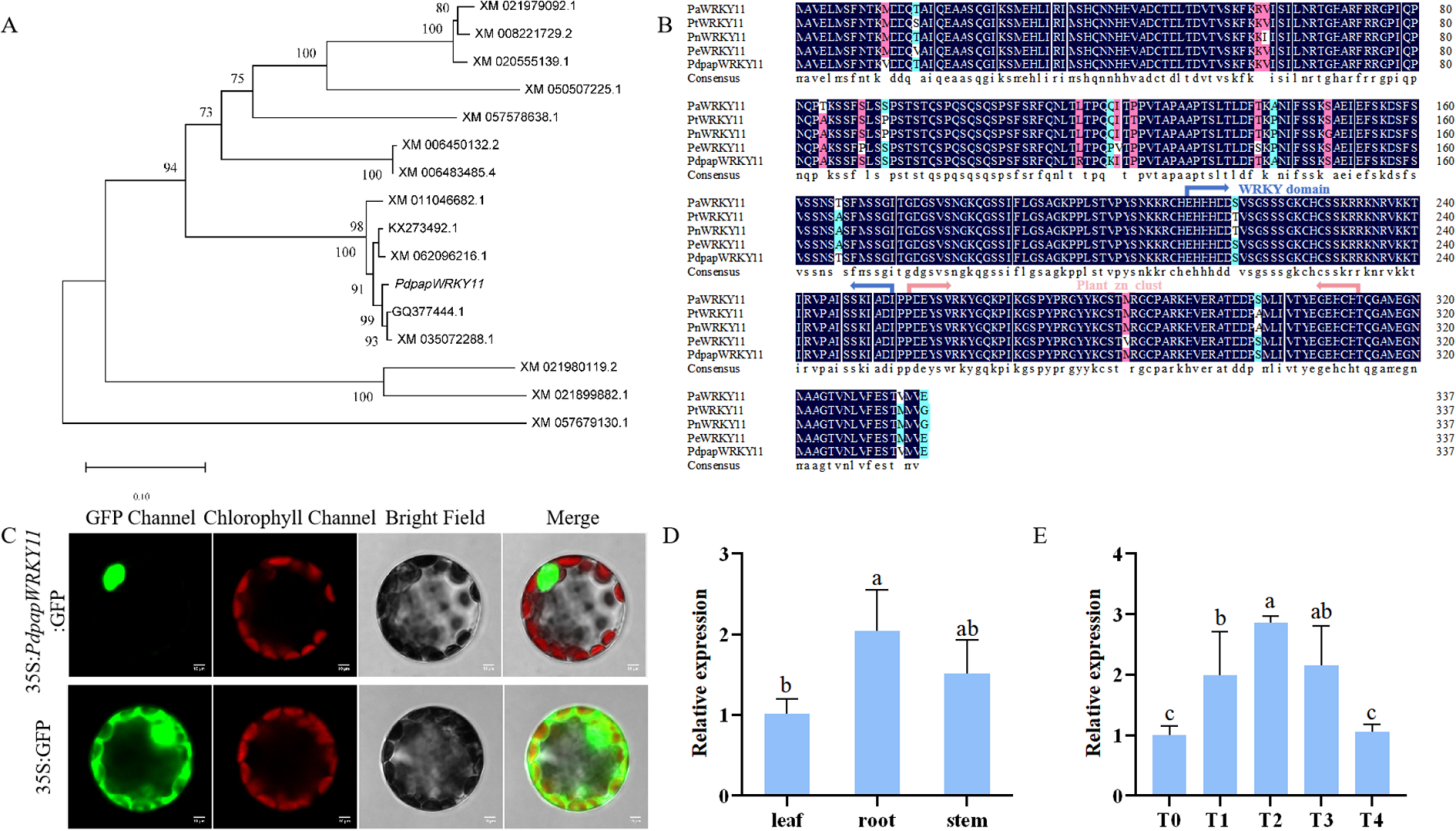
The characterization of *PdpapWRKY11*. **(A)** Phylogenetic tree of WRKY11 protein in different species. **(B)** Multiple alignment of the amino acid sequences of WRKY11 proteins from *Populus alba*, *P. trichocarpa*, *P. nigra*, and *P. euphratica* and *PdpapWRKY11*. **(C)** Subcellular localization of *PdpapWRKY11*. The GFP was used as the control. **(D)** The RT-qPCR detection results of *PdpapWRKY11* in stem, root and leaf. **(E)** The RT-qPCR analysis of the *PdpapWRKY11* expression under *Botryosphaeria dothidea* stress with *PdpapEF1-α* as internal controls. The 2^−ΔΔCT^ method was used for quantification. T0, T1, T2, T3 and T4 indicated that plants were subjected to stress treatment for 0, 6, 12, 24 and 48 h, respectively. Error bars indicate the standard deviation of three independent biological replicates. Significant differences between groups (*P* < 0.05), determined by Student’s t-test, are denoted by different lowercase letters.

Our research group previously conducted transcriptome sequencing on Pdpap infected with *B. dothidea* at five critical timepoints (0, 6, 12, 24, and 48 h) to investigate the resistance mechanisms of Pdpap against the pathogen. Based on RNA-seq analysis using thresholds of |log_2_FC| > 2 and FDR< 0.01, four genes were persistently up-regulated and three genes consistently showed down-regulated trends across all treatment groups compared to 0 h Pdpap seedlings (**Supplementary Figure 4**, **Supplementary Table 8**). Among the four up-regulated genes, only *PdpapWRKY11* belongs to Subgroup IId of *P. trichocarpa* (previously identified as a stress-responsive subfamily). Among these, *PdpapWRKY11* (homologous to the stress-responsive subfamily IId of *P. trichocarpa*) was thus selected for subsequent experiments. The *PdpapWRKY11* gene was cloned from PdPap. The sequence contains a 1,017 bp open reading frame (ORF) encoding 338 amino acids. Phylogenetic analysis revealed high homology between *PdpapWRKY11* and WRKY11 sequences from *P. alba*, *P. trichocarpa*, *P. nigra*, and *P. euphratica* (**Figure 3A**). Multiple sequence alignment of *PaWRKY11*, *PtWRKY11*, *PnWRKY11*, *PeWRKY11*, and *PdpapWRKY11* showed 98.66% identity, with the highest similarity to *P. alba*. Conserved domain analysis confirmed the presence of WRKY and Plant_zn_clust domains (**Figure 3B**).

To determine the intracellular localization of *PdpapWRKY11*, the pBWA(V)HS-GFP vector was digested with EcoRV and ligated to *PdpapWRKY11* via homologous recombination. GFP fluorescence, visualized under a confocal laser scanning microscope, confirmed nuclear localization of the pBWA(V)HS-*PdpapWRKY11*-GFP fusion protein in transfected *Arabidopsis* protoplasts (**Figure 3C**).

Tissue-specific expression analysis revealed that *PdpapWRKY11* transcripts were most abundant in roots (2.02-fold higher than leaves) and least in leaves, with intermediate levels in stems (1.35-fold higher than leaves)(**Figure 3D**). Under *B. dothidea* infection, *PdpapWRKY11* expression was significantly induced, peaking at 12 h (2.86-fold higher than 0 h) and declining to 2.15-fold by 24 h (**Figure 3E**). All infected groups showed markedly higher expression than the 0 h control, confirming *B. dothidea* responsive activation of *PdpapWRKY11*.

**Figure 4:**
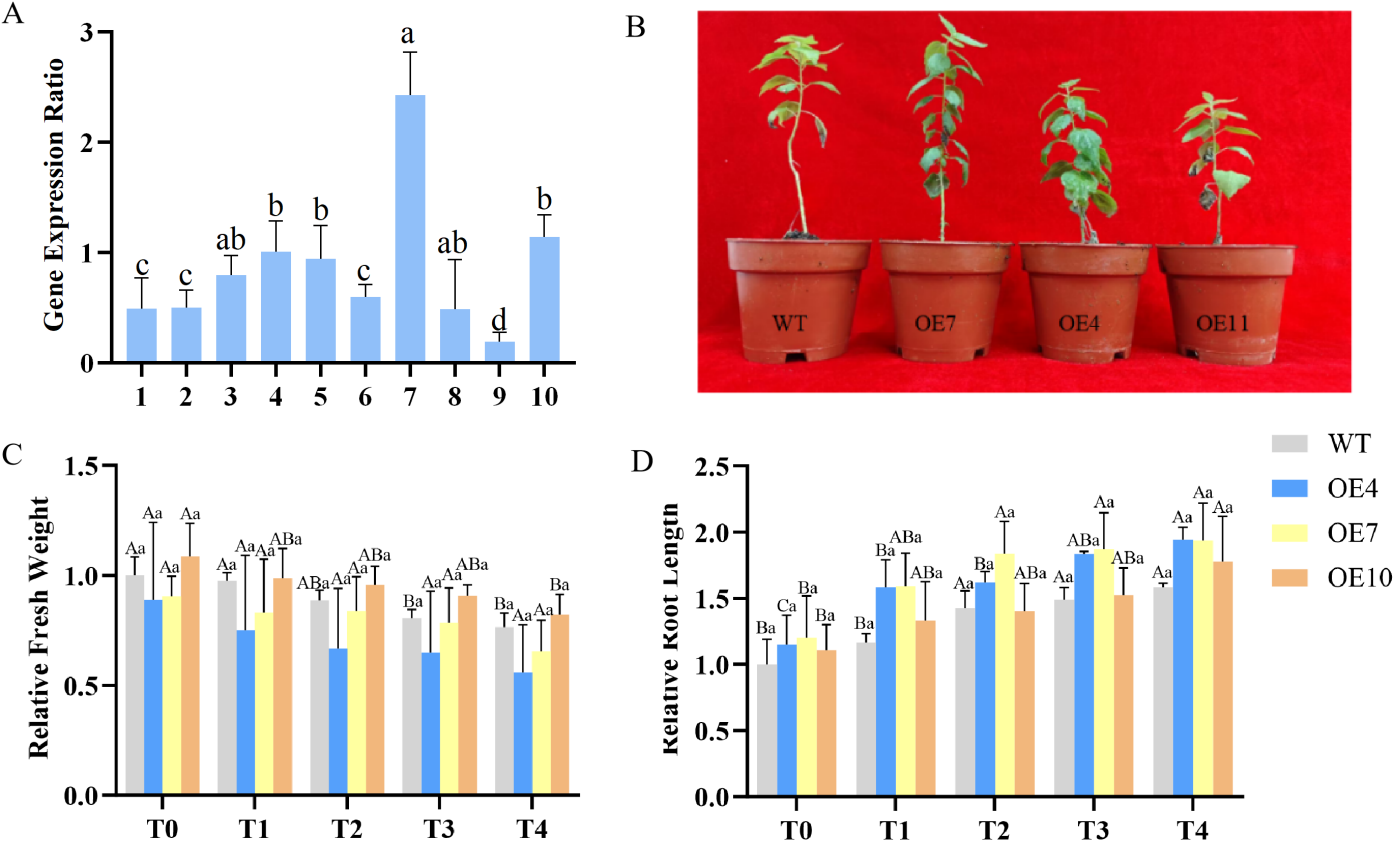
Plant status and growth indicators. **(A)** Expression level analysis of *PdpapWRKY11* in transformants with *PdpapEF1-α* as internal controls. The 2^−ΔΔCT^ method was used for quantification. **(B)** The growth status of wild *P. davidiana* × *P. alba* var. *Pyramidalis* and overexpressing transformants OE4, OE7, OE10 lines infected by *Botryosphaeria dothidea* and grown for 20 **(C, D)** Relative fresh weight of wild Pdpap and transformants treated with *B. dothidea*. **(D)** Root lengths of wild Pdpap and transformants treated with *B. dothidea*. T0–T4: Infection times were 0, 5, 10, 15 and 20 **(D)** The error bars represent standard deviation of three independent replicates. Error bars represent the standard deviation of three independent biological replicates. Significant differences (*P<* 0.05) were determined by two-way ANOVA, with between-group differences indicated by different uppercase letters and within-group differences indicated by different lowercase letters.

Ten *PdpapWRKY11* transformant Pdpap lines were generated via Agrobacterium-mediated transformation. PCR with gene-specific and vector primers (*PdpapWRKY11*-F*/*pBI121-R, *PdpapWRKY11*-R*/*pBI121-F) confirmed transgene integration in all lines, with bands matching the positive control (pBI121-*PdpapWRKY11*) and negative controls (water) or no amplification in WT (**Supplementary Figure 5**). qRT-PCR identified OE4, OE7, and OE10 as high-expressing lines, while other lines showed suboptimal expression. RNA-based PCR validation confirmed overexpression in all 10 lines, with OE4, OE7, and OE10 selected for subsequent studies (**Figure 4A**).

To investigate the role of *PdpapWRKY11* in Pdpap’s response to *B. dothidea*, transformant Pdpap lines (OE4, OE7, OE10) and WT seedlings were inoculated with the pathogen. As shown in **Figure 4B**, WT seedlings exhibited severe leaf abscission and stem lesions after inoculation, while OE lines maintained healthy growth (the pathological manifestations are shown in **Supplementary Figure 6**), indicating that *PdpapWRKY11* overexpression enhances resistance to *B. dothidea*. Statistical analysis of disease indices revealed that the WT exhibited a significantly higher disease index (83%) compared to the transformant lines (20%).

Fresh weight analysis (**Figure 4C**) revealed no significant changes in OE4 and OE7 during T0-T4, whereas WT showed significant reductions at all time points. OE10 displayed significant fresh weight changes only during T0-T1 and T3-T4. Root length remained stable across all lines (**Figure 4D**), but OE plants exhibited superior growth under pathogen stress. These results demonstrate that *PdpapWRKY11* overexpression mitigates the negative impact of *B. dothidea* on seedling growth.

The antioxidant role of *PdpapWRKY11* was analyzed by measuring antioxidant enzyme activities (POD, T-SOD) and oxidative damage markers (H_2_O_2_, MDA). Under *B. dothidea* stress, all lines showed an initial increase followed by a decline in POD and T-SOD activities. During early infection (T0-T1), OE lines exhibited significantly higher POD (16.92–18.64% increase) and T-SOD (10.73–19.46% increase) activities than WT (**Supplementary Figures 7A, B**).

H_2_O_2_ levels exhibited stage-specific regulation patterns (**Supplementary Figure 7C**). Under *B. dothidea* stress, all lines showed a dynamic “initial decrease followed by increase” in H_2_O_2_ content. At T0, H_2_O_2_ levels in OE4 and OE7 showed significantly lower levels. By T1, WT and OE10 accumulated more H_2_O_2_ than OE4 and OE7. At T2, the transformant Pdpap lines transiently surpassed WT in H_2_O_2_ accumulation. During T3-T4, H_2_O_2_ levels in OE lines were significantly lower than in WT. These results indicate that *PdpapWRKY11* dynamically regulates reactive oxygen species (ROS) homeostasis to balance disease resistance responses.

MDA levels followed a similar “initial decrease followed by increase” trend as H_2_O_2_ (**Supplementary Figure 7D**). During early infection (T0), MDA content in OE lines was 20.27-96.72% lower than in WT. However, by T3, OE7 showed significantly higher MDA levels than WT, potentially linked to stage-specific lipid peroxidation regulation. These findings demonstrate that *PdpapWRKY11* enhances systemic resistance against canker disease by temporally regulating antioxidant enzyme activities and oxidative damage markers.

### 3.3 *PdpapWRKY11* overexpression enhances lignin biosynthesis in Pdpap

**Figure 5:**
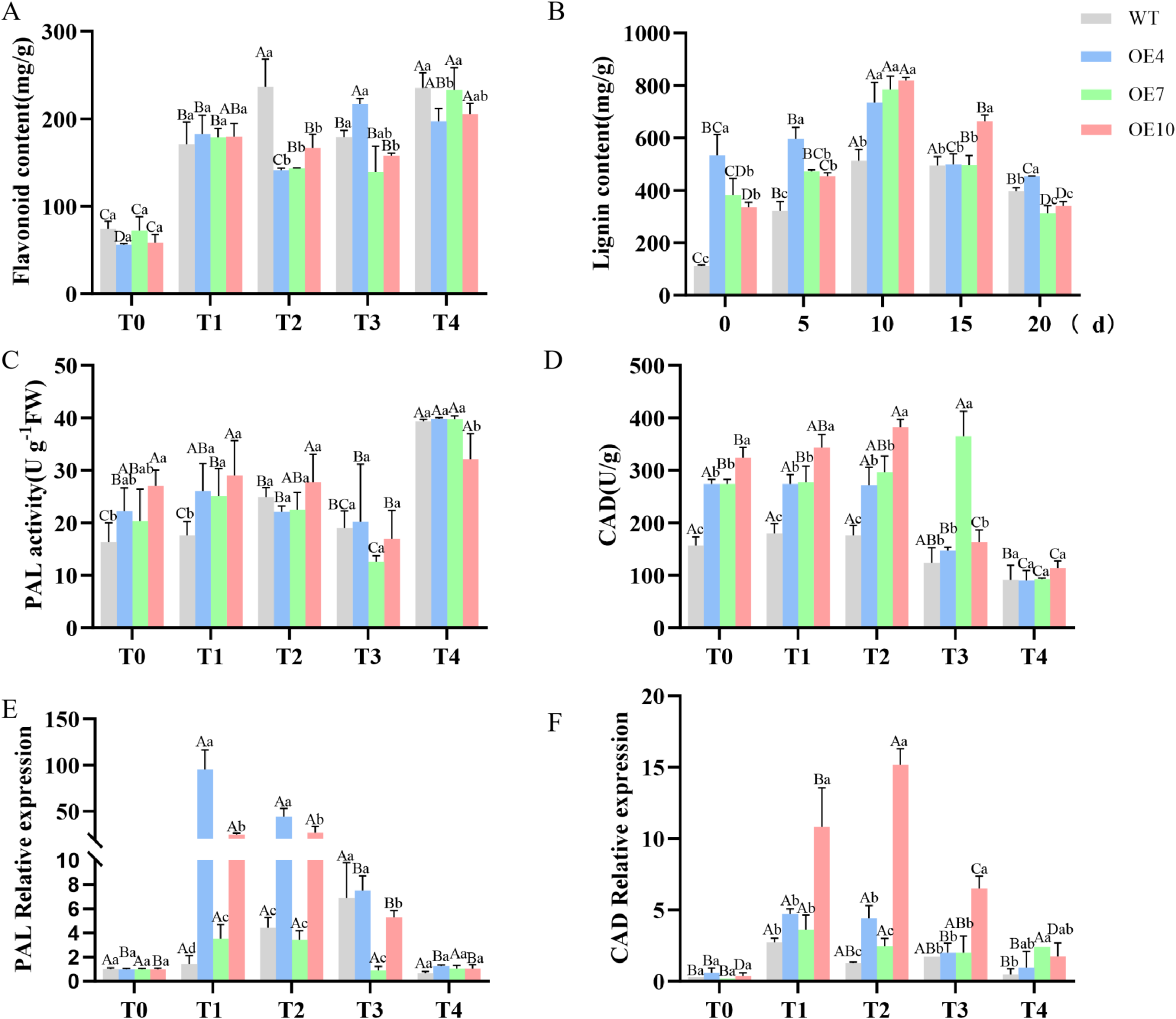
Determination of enzyme activities and key genes in lignin biosynthesis pathway. **(A)** Quantification results of lignin content via acetyl bromide method; **(B)** Quantification results of flavonoid content via colorimetric assay; **(C)** Quantification results of PAL activity via colorimetric assay; **(D)** Quantification results of CAD activity via NADH-dependent activity assay; **(E)** The RT-qPCR analysis of the *PdpapPAL* expression under *Botryosphaeria dothidea* stress with *PdpapEF1-α* as internal controls; **(F)** The RT-qPCR analysis of the *PdpapCAD* expression under *B.dothidea* stress with *PdpapEF1-α* as internal controls. The 2^−ΔΔCT^ method was used for quantification. T0−T4: Infection times were 0, 6, 12, 24, 48 **(H)** Error bars represent the standard deviation of three independent biological replicates. Significant differences (*P<* 0.05) were determined by two-way ANOVA, with between-group differences indicated by different uppercase letters and within-group differences indicated by different lowercase letters.

To investigate the metabolic regulatory role of *PdpapWRKY11* in Pdpap’s defense against *B. dothidea*, lignin and flavonoid accumulation patterns were systematically analyzed in transformant Pdpap lines (OE4, OE7, OE10) and WT plants. Under pathogen stress, all lines exhibited a dynamic “initial increase followed by decrease” in lignin and flavonoid content. During early infection (0–10 d), OE lines accumulated significantly more lignin than WT. At 10 d post-inoculation, lignin levels in OE lines were 30.20–37.43% higher than in WT (*P* < 0.01). In later stages (10–20 d), lignin content declined in all lines (**Figure 5A**). Flavonoid levels showed no significant differences between OE and WT during T0-T1. However, during T2-T4, WT exhibited 29.26–40.00% higher flavonoid accumulation than OE lines (*P* < 0.01) (**Figure 5B**). These results suggest that *PdpapWRKY11* preferentially enhances lignin biosynthesis, which plays a critical role in defense against *B. dothidea*.

To elucidate how *PdpapWRKY11* regulates lignin biosynthesis under *B. dothidea* stress, the activities of key phenylpropanoid pathway enzymes (PAL and CAD) and related gene expression were systematically measured. Under pathogen stress, all lines showed an “initial increase followed by decrease” in PAL and CAD activities. During T0-T1, PAL activity in OE lines was significantly higher than in WT, with a 30.04–39.58% increase at T1. CAD activity remained elevated in OE lines compared to WT during T0-T2 (**Figures 5C, D**).

qRT-PCR results confirmed significant upregulation of *PdpapPAL* and *PdpapCAD* in OE lines, consistent with enzyme activity trends (**Figures 5E, F**). These findings indicate that *PdpapWRKY11* may activate *PdpapPAL* and *PdpapCAD* to enhance lignin biosynthesis, thereby strengthening pathogen defense.

## 4 Discussion

The WRKY TFs family, serving as a central regulatory hub in plant defense against biotic stress, has established a relatively comprehensive theoretical framework for its functional differentiation and evolutionary mechanisms in herbaceous plants. However, research on WRKY families in woody species remains incomplete due to their genomic complexity. Studies have revealed significant diversity in WRKY gene family sizes across species, with varying numbers identified in *A. thaliana* (Grzechowiak et al., 2022), *Zea mays* (Wang et al., 2023), *Cucurbita pepo* (Chen et al., 2024), *Vitis vinifera* (Vodiasova et al., 2024), *Vaccinium* sp (Lei et al., 2024), *Mikania micrantha* (Zhang et al., 2025), and *Gentiana macrophylla* (Gu et al., 2025).

This study systematically elucidates the evolutionary dynamics and functional architecture of *PtrWRKYs* in *P. trichocarpa*, providing novel insights into the adaptive evolution of transcriptional regulatory networks in woody plants. This study classified the identified 102 *PtrWRKYs* into three major clades and five subclades through phylogenetic analysis. The absence of WRKY genes on chromosome Chr09 is consistent with reports of evolutionarily conserved selective gene loss in plants (Wu et al., 2014; Jiang et al., 2014).

However, Jiang identified only 100 *PtrWRKYs* and limited their family analysis to phylogenetic tree construction and promoter cis-element prediction. Building on this foundation, our research supplements two additional WRKY family members and extends analytical dimensions to include motif architecture, gene structure, chromosomal localization, syntenic relationships, and protein structure with subcellular localization, thus enhancing the understanding of the *P. trichocarpa* WRKY gene family. Gene duplication types and selective pressures are identified as key evolutionary forces driving functional diversification. The expansion of PtrWRKYs is predominantly driven by segmental duplication events (95 pairs, Ka/Ks=0.24, purifying selection), a pattern consistent with the segmental duplication-dominated expansion strategies observed in *Arabidopsis*, peanut, and other *Populus* species (Song et al., 2016). This segmental duplication-dominant mode likely reflects the evolutionary demands of woody plants adapting to complex ecological environments while avoiding genomic instability potentially induced by tandem duplication. Additionally, the nuclear localization of most PtrWRKYs (94.1%) aligns with their canonical transcriptional regulatory roles. Notably, conserved motif compositions, domain architectures, and gene structures within subgroups, such as the retention of dual WRKY domains in Group I versus single domains in Group II/III, alongside the unique presence of the Plant_zn_clust domain in Subgroup IId, suggest functional coherence within subgroups. The dual WRKY domains in Group I may enhance regulatory specificity through cooperative DNA binding or protein interactions, while single domains in Group II/III likely enable functional innovation through cis-regulatory element variation (Javed and Gao, 2023). Intriguingly, the acquisition of the Plant_zn_clust domain (a zinc-binding motif linked to stress adaptation and developmental regulation) in Subgroup IId mirrors functional modularization strategies observed in WRKY families of rice, tomato, and peanut (Javed and Gao, 2023). Collectively, this study demonstrates that functional diversification in woody plant WRKY families is achieved through segmental duplication, domain architecture remodeling, and cis-regulatory evolution, laying a theoretical foundation for future applications of WRKY family genes in genetic improvement strategies.

By integrating genome-wide identification of *PtrWRKYs* and the functional validation system in Pdpap, this study clarified the molecular pathway by which *PdpapWRKY11*, a member of the IId subgroup of the PtrWRKY family, enhances poplar resistance to *B. dothidea* through mediating lignin biosynthesis. Based on RNA-seq analysis, we identified the *PdpapWRKY11*, which is homologous to *PtrWRKY93* from the stress-responsive Subgroup IId of *P. trichocarpa*. This gene exhibited significant upregulation during *B. dothidea* infection and was selected for functional validation. Through constructing transformant Pdpap lines (OE4, OE7, OE10), we found they exhibited significantly enhanced resistance to *B. dothidea* infection compared to WT (*P<* 0.01). Previous studies have demonstrated the evolutionary conservation of WRKY11 homologs in plant stress responses. Under abiotic stress, *MsWRKY11* from *Medicago sativa* enhances salt tolerance by activating ion homeostasis-related genes (Wang et al., 2018). while heterologous expression of *PcWRKY11* from *Polygonum cuspidatum* in *A. thaliana* significantly improves salt stress resilience (Wang GW, et al., 2022). In biotic stress contexts, *OsWRKY11* in *Oryza sativa* confers pathogen resistance through constitutive activation of defense-related genes (Lee et al., 2018), and studies in *N. tabacum*-*Ralstonia solanacearum* and *Brassica napus*-*Xanthomonas campestris* systems further validate the positive regulatory role of WRKY11 in disease signaling (Li et al., 2021; Yang et al., 2022). These findings provide a theoretical framework for elucidating *PdpapWRKY11* functionality. Simultaneously, we observed that transformant Pdpap lines showed significantly reduced fresh weight loss post-pathogen infection (*P* < 0.01), though root length showed no statistical difference. This phenotypic divergence may correlate with *B. dothidea*-induced carbon starvation: fungal infection reduced net photosynthetic rate (Pn) and triggered hydraulic failure-induced leaf abscission due to elevated xylem water potential near canker lesions (Xing et al., 2020). The core of plant immunity lies in the dynamic balance of ROS homeostasis. Our results reveal that OE lines enhance early defense via temporally regulated antioxidant enzymes: at 24 h, POD and T-SOD activities peaked higher than WT, respectively, delaying H_2_O_2_ burst timing. Oxidative damage markers further indicated that MDA content in OE lines decreased by 20.27%-96.72% at 0 h compared to WT, while OE7 showed a marked MDA increase at 24 h, suggesting *PdpapWRKY11* involvement in lipid peroxidation feedback regulation (Wang G. W. et al., 2022). Based on integrated phenotypic and physiological analyses of *B. dothidea*-infected Pdpap, OE lines exhibited significantly reduced oxidative damage during late infection stages. This dual-phase resistance mechanism likely involves: early activation of antioxidant genes for rapid ROS scavenging, and synergy with the NPR1-dependent SA pathway to maintain systemic acquired resistance (SAR), thereby curbing oxidative damage propagation. Additionally, expression variations of *PdpapWRKY11* observed in transformant Pdpap lines (OE4, OE7, OE10) may stem from genomic position effects and T-DNA insertion copy number variations. The nuclear localization of *PdpapWRKY11* likely interfered with qRT-PCR accuracy, necessitating post-RNA extraction PCR validation to confirm overexpression. These results demonstrate *PdpapWRKY11* plays a critical role in maintaining physiological homeostasis post-infection.

During *B. dothidea* infection, *PdpapWRKY11* orchestrates a time-partitioned defense strategy by differentially regulating lignin (0–20 d) and flavonoid (0–48 h) accumulation-a hallmark of woody plant adaptation. Overexpression boosted lignin levels by 30.20%-37.43% in OE lines versus WT at 10 d, while flavonoid accumulation in WT surpassed OE lines by 29.26-40.00% at 12 h. These results imply that sustained lignin precursor synthesis may compete for shared phenylpropanoid pathway substrates, suggesting a potential “substrate competition” relationship between the lignin and flavonoid branches of the phenylpropanoid pathway.

This study has elucidated the disease resistance function and regulatory mechanism of *PdpapWRKY11*. However, several aspects merit further investigation: Future research should focus on identifying upstream regulators and downstream targets of *PdpapWRKY11* to refine the regulatory network it mediates, while simultaneously exploring in depth the regulatory mechanism of *PdpapWRKY11* on lignin monomer composition. These efforts will provide comprehensive genetic resources and theoretical frameworks for disease-resistant poplar breeding.

## 5 Conclusion

In this study, we identified 102 *PtrWRKYs* from the *P. trichocarpa* genome, classified them into 7 subclades based on their phylogenetic relationships, and analyzed their gene structures, conserved motifs, chromosomal localization, collinearity, and cis-acting elements in the promoter regions. Notably, the promoter regions of PtrWRKYs contain stress-responsive elements. To elucidate the role of the WRKY family in poplar resistance to biotic stress, we selected Pdpap, which exhibits stronger resistance to *B. dothidea*, as the research subject. Additionally, we cloned a transcription factor, *PdpapWRKY11* from Pdpap and found that *PdpapWRKY11* is localized in the nucleus. Transformant Pdpap lines overexpressing *PdpapWRKY11* showed significantly enhanced resistance to *B. dothidea*. Further investigations revealed that *PdpapWRKY11* regulates lignin accumulation by enhancing the activities of phenylpropanoid pathway enzymes, PAL and CAD. This enhancement may be attributed to its activation of the corresponding genes, *PdpapPAL* and *PdpapCAD*. Therefore, we conclude that *PdpapWRKY11* plays a critical regulatory role in the stress response to *B. dothidea*. However, the response mechanisms of *PtrWRKYs* to biotic stress remain to be further elucidated.

## Data Availability

The datasets presented in this study can be found in online repositories. The names of the repository/repositories and accession number(s) can be found in the article/**Supplementary Material**.
