# Supplementary material for: Enhanced resistance to Botryosphaeria dothidea through upregulation of the lignin biosynthesis regulator WRKY11 in poplar
Source: Front Plant Sci. 2026 Feb 26;17:1737207. doi: 10.3389/fpls.2026.1737207 (PMC12979517; doi:10.3389/fpls.2026.1737207)
Supplement: Supplementary file 4 [file Table4.docx]

**Table S4**. Predition of the Sencondary Structure in PtrWRKY Proteins

| Name in this paper | Locus tag | Number of Alpha helix | The percent of all the sencondary structures | Number of Extended strand | The percent of all the sencondary structures | Number of Random coil | The percent of all the sencondary structures |
| --- | --- | --- | --- | --- | --- | --- | --- |
| PtrWRKY1 | Potri.001G002400.1.v3.0 | 10 | 7.41% | 14 | 10.37% | 111 | 82.22% |
| PtrWRKY2 | Potri.001G044500.1.v3.0 | 67 | 21.07% | 29 | 9.12% | 222 | 69.81% |
| PtrWRKY3 | Potri.001G058800.1.v3.0 | 27 | 16.88% | 16 | 10.00% | 117 | 73.12% |
| PtrWRKY4 | Potri.001G092900.1.v3.0 | 63 | 18.64% | 23 | 6.80% | 252 | 74.56% |
| PtrWRKY5 | Potri.001G099000.1.v3.0 | 56 | 17.23% | 16 | 4.92% | 253 | 77.85% |
| PtrWRKY6 | Potri.001G121300.1.v3.0 | 58 | 18.47% | 23 | 7.32% | 233 | 74.20% |
| PtrWRKY7 | Potri.001G208600.1.v3.0 | 107 | 20.62% | 34 | 6.55% | 378 | 72.83% |
| PtrWRKY8 | Potri.001G328000.1.v3.0 | 33 | 12.36% | 18 | 6.74% | 216 | 80.90% |
| PtrWRKY9 | Potri.001G352400.1.v3.0 | 30 | 9.62% | 15 | 4.81% | 267 | 85.58% |
| PtrWRKY10 | Potri.001G361600.1.v3.0 | 31 | 5.56% | 40 | 7.17% | 487 | 87.28% |
| PtrWRKY11 | Potri.001G460600.1.v3.0 | 61 | 14.42% | 26 | 6.15% | 336 | 79.43% |
| PtrWRKY12 | Potri.001G472800.1.v3.0 | 46 | 6.28% | 41 | 5.60% | 645 | 88.11% |
| PtrWRKY13 | Potri.002G043500.1.v3.0 | 55 | 15.67% | 15 | 4.27% | 281 | 80.06% |
| PtrWRKY14 | Potri.002G059100.1.v3.0 | 25 | 7.69% | 17 | 5.23% | 283 | 87.08% |
| PtrWRKY15 | Potri.002G123300.1.v3.0 | 69 | 19.27% | 19 | 5.31% | 270 | 75.42% |
| PtrWRKY16 | Potri.002G138900.1.v3.0 | 21 | 10.34% | 19 | 94.36% | 163 | 80.30% |
| PtrWRKY17 | Potri.002G164400.1.v3.0 | 60 | 16.95% | 16 | 4.52% | 278 | 78.53% |
| PtrWRKY18 | Potri.002G164900.1.v3.0 | 30 | 15.54% | 17 | 8.81% | 146 | 75.65% |
| PtrWRKY19 | Potri.002G168700.1.v3.0 | 63 | 17.36% | 17 | 4.68% | 283 | 77.96% |
| PtrWRKY20 | Potri.002G186600.1.v3.0 | 105 | 20.75% | 40 | 7.91% | 361 | 71.34% |
| PtrWRKY21 | Potri.002G193000.1.v3.0 | 34 | 10.73% | 15 | 4.73% | 268 | 84.54% |
| PtrWRKY22 | Potri.002G195300.2.v3.0 | 45 | 18.37% | 14 | 5.71% | 186 | 75.92% |
| PtrWRKY23 | Potri.002G221600.1.v3.0 | 26 | 7.07% | 35 | 9.51% | 307 | 83.42% |
| PtrWRKY24 | Potri.002G228400.1.v3.0 | 117 | 18.96% | 41 | 6.65% | 459 | 74.39% |
| PtrWRKY25 | Potri.003G111900.1.v3.0 | 56 | 18.06% | 20 | 6.45% | 234 | 75.48% |
| PtrWRKY26 | Potri.003G132700.1.v3.0 | 57 | 17.27% | 16 | 4.85% | 257 | 77.88% |
| PtrWRKY27 | Potri.003G138600.1.v3.0 | 61 | 17.84% | 16 | 4.68% | 265 | 77.49% |
| PtrWRKY28 | Potri.003G169100.1.v3.0 | 26 | 16.56% | 16 | 10.19% | 115 | 73.25% |
| PtrWRKY29 | Potri.003G182200.2.v3.0 | 94 | 29.47% | 30 | 9.40% | 195 | 61.13% |
| PtrWRKY30 | Potri.004G007500.1.v3.0 | 117 | 20.24% | 44 | 7.61% | 417 | 72.15% |
| PtrWRKY31 | Potri.004G060400.1.v3.0 | 39 | 14.89% | 17 | 6.49% | 206 | 78.63% |
| PtrWRKY32 | Potri.004G060900.1.v3.0 | 39 | 14.89% | 17 | 6.49% | 206 | 78.63% |
| PtrWRKY33 | Potri.004G072000.1.v3.0 | 54 | 12.56% | 15 | 3.49% | 361 | 83.95% |
| PtrWRKY34 | Potri.004G120800.1.v3.0 | 27 | 4.97% | 39 | 7.18% | 477 | 87.85% |
| PtrWRKY35 | Potri.005G055300.1.v3.0 | 54 | 15.30% | 13 | 3.68% | 286 | 81.02% |
| PtrWRKY36 | Potri.005G085200.1.v3.0 | 15 | 7.28% | 19 | 9.22% | 172 | 83.50% |
| PtrWRKY37 | Potri.005G086400.1.v3.0 | 29 | 13.00% | 17 | 7.62% | 177 | 79.37% |
| PtrWRKY38 | Potri.005G141400.1.v3.0 | 66 | 19.94% | 18 | 5.44% | 247 | 74.62% |
| PtrWRKY39 | Potri.005G203200.1.v3.0 | 21 | 6.52% | 17 | 5.28% | 284 | 88.20% |
| PtrWRKY40 | Potri.005G219500.1.v3.0 | 53 | 15.27% | 15 | 4.32% | 279 | 80.40% |
| PtrWRKY41 | Potri.006G072400.1.v3.0 | 59 | 19.60% | 15 | 4.98% | 227 | 75.42% |
| PtrWRKY42 | Potri.006G087000.1.v3.0 | 36 | 11.84% | 24 | 7.89% | 244 | 80.26% |
| PtrWRKY43 | Potri.006G105300.1.v3.0 | 33 | 6.04% | 43 | 7.88% | 470 | 86.08% |
| PtrWRKY44 | Potri.006G109100.1.v3.0 | 64 | 19.22% | 16 | 4.80% | 253 | 75.98% |
| PtrWRKY45 | Potri.006G133200.8.v3.0 | 24 | 5.05% | 32 | 6.74% | 419 | 88.21% |
| PtrWRKY46 | Potri.006G184800.1.v3.0 | 47 | 9.00% | 36 | 6.90% | 439 | 84.10% |
| PtrWRKY47 | Potri.006G224100.1.v3.0 | 19 | 11.52% | 17 | 10.30% | 129 | 78.18% |
| PtrWRKY48 | Potri.006G263600.1.v3.0 | 88 | 27.50% | 26 | 8.12% | 206 | 64.38% |
| PtrWRKY49 | Potri.006G264000.1.v3.0 | 40 | 8.39% | 35 | 7.34% | 402 | 84.28% |
| PtrWRKY50 | Potri.007G047400.1.v3.0 | 64 | 19.10% | 22 | 6.57% | 249 | 74.33% |
| PtrWRKY51 | Potri.007G078200.1.v3.0 | 17 | 7.30% | 19 | 8.15% | 197 | 84.55% |
| PtrWRKY52 | Potri.007G079800.1.v3.0 | 16 | 8.33% | 19 | 9.90% | 157 | 81.77% |
| PtrWRKY53 | Potri.008G091900.1.v3.0 | 24 | 4.88% | 43 | 8.74% | 425 | 86.38% |
| PtrWRKY54 | Potri.008G094000.1.v3.0 | 20 | 6.83% | 15 | 5.12% | 258 | 88.05% |
| PtrWRKY55 | Potri.008G103300.1.v3.0 | 44 | 11.96% | 21 | 5.71% | 303 | 82.34% |
| PtrWRKY56 | Potri.010G147700.1.v3.0 | 50 | 13.37% | 22 | 5.88% | 302 | 80.75% |
| PtrWRKY57 | Potri.010G160100.2.v3.0 | 27 | 9.22% | 21 | 7.17% | 245 | 83.62% |
| PtrWRKY58 | Potri.010G163000.1.v3.0 | 29 | 5.81% | 33 | 6.61% | 437 | 87.58% |
| PtrWRKY59 | Potri.011G007800.1.v3.0 | 120 | 20.24% | 43 | 7.25% | 430 | 72.51% |
| PtrWRKY60 | Potri.011G070100.1.v3.0 | 38 | 14.18% | 15 | 5.60% | 215 | 80.22% |
| PtrWRKY61 | Potri.011G079300.1.v3.0 | 23 | 7.52% | 15 | 4.90% | 268 | 87.58% |
| PtrWRKY62 | Potri.011G087900.1.v3.0 | 18 | 3.21% | 41 | 7.31% | 502 | 89.48% |
| PtrWRKY63 | Potri.011G157100.1.v3.0 | 48 | 10.67% | 19 | 4.22% | 383 | 85.11% |
| PtrWRKY64 | Potri.011G169300.1.v3.0 | 32 | 4.41% | 42 | 5.79% | 651 | 89.79% |
| PtrWRKY65 | Potri.012G031700.1.v3.0 | 67 | 18.06% | 19 | 5.12% | 285 | 76.82% |
| PtrWRKY66 | Potri.012G101000.1.v3.0 | 16 | 8.60% | 18 | 9.68% | 152 | 81.72% |
| PtrWRKY67 | Potri.013G042600.1.v3.0 | 51 | 14.41% | 15 | 4.24% | 288 | 81.36% |
| PtrWRKY68 | Potri.013G086000.1.v3.0 | 39 | 5.45% | 39 | 5.45% | 638 | 89.11% |
| PtrWRKY69 | Potri.013G090300.1.v3.0 | 76 | 23.46% | 19 | 5.86% | 229 | 70.68% |
| PtrWRKY70 | Potri.013G090400.1.v3.0 | 101 | 28.69% | 24 | 6.82% | 227 | 64.49% |
| PtrWRKY71 | Potri.013G153400.1.v3.0 | 32 | 5.41% | 39 | 6.60% | 520 | 87.99% |
| PtrWRKY72 | Potri.014G009500.1.v3.0 | 32 | 18.82% | 12 | 7.06% | 126 | 74.12% |
| PtrWRKY73 | Potri.014G024200.1.v3.0 | 75 | 19.33% | 17 | 4.38% | 296 | 76.29% |
| PtrWRKY74 | Potri.014G050000.1.v3.0 | 19 | 8.33% | 20 | 8.77% | 189 | 82.89% |
| PtrWRKY75 | Potri.014G090300.1.v3.0 | 61 | 17.48% | 16 | 4.58% | 272 | 77.94% |
| PtrWRKY76 | Potri.014G090700.1.v3.0 | 32 | 16.93% | 16 | 8.47% | 141 | 74.60% |
| PtrWRKY77 | Potri.014G096200.1.v3.0 | 68 | 18.63% | 18 | 4.93% | 279 | 76.44% |
| PtrWRKY78 | Potri.014G111900.1.v3.0 | 115 | 22.91% | 39 | 7.77% | 348 | 69.32% |
| PtrWRKY79 | Potri.014G118200.1.v3.0 | 30 | 9.40% | 20 | 6.27% | 269 | 84.33% |
| PtrWRKY80 | Potri.014G119800.1.v3.0 | 37 | 14.12% | 16 | 6.11% | 209 | 79.77% |
| PtrWRKY81 | Potri.014G155100.1.v3.0 | 126 | 20.19% | 50 | 8.01% | 448 | 71.79% |
| PtrWRKY82 | Potri.014G164300.1.v3.0 | 20 | 4.12% | 32 | 6.60% | 433 | 89.28% |
| PtrWRKY83 | Potri.015G064100.1.v3.0 | 119 | 18.95% | 36 | 5.73% | 473 | 75.32% |
| PtrWRKY84 | Potri.015G099200.1.v3.0 | 25 | 14.04% | 18 | 10.11% | 135 | 75.84% |
| PtrWRKY85 | Potri.016G083600.5.v3.0 | 26 | 5.54% | 30 | 6.40% | 413 | 88.06% |
| PtrWRKY86 | Potri.016G099900.1.v3.0 | 39 | 12.62% | 23 | 7.44% | 247 | 79.94% |
| PtrWRKY87 | Potri.016G128300.1.v3.0 | 24 | 4.15% | 37 | 6.39% | 518 | 89.46% |
| PtrWRKY88 | Potri.016G137900.1.v3.0 | 58 | 18.07% | 21 | 6.54% | 242 | 75.39% |
| PtrWRKY89 | Potri.017G079500.1.v3.0 | 103 | 19.14% | 33 | 6.13% | 402 | 74.72% |
| PtrWRKY90 | Potri.017G088300.1.v3.0 | 36 | 6.73% | 43 | 8.04% | 456 | 85.23% |
| PtrWRKY91 | Potri.017G104800.1.v3.0 | 34 | 14.35% | 15 | 6.33% | 188 | 79.32% |
| PtrWRKY92 | Potri.017G149000.1.v3.0 | 45 | 10.92% | 19 | 4.61% | 348 | 84.47% |
| PtrWRKY93 | Potri.018G008500.1.v3.0 | 59 | 17.46% | 18 | 5.33% | 261 | 77.22% |
| PtrWRKY94 | Potri.018G019000.1.v3.0 | 45 | 9.66% | 30 | 6.44% | 391 | 83.91% |
| PtrWRKY95 | Potri.018G019700.1.v3.0 | 79 | 29.15% | 22 | 8.12% | 170 | 62.73% |
| PtrWRKY96 | Potri.018G019800.1.v3.0 | 84 | 26.25% | 31 | 9.69% | 205 | 64.06% |
| PtrWRKY97 | Potri.018G107000.1.v3.0 | 71 | 13.37% | 31 | 5.84% | 429 | 80.79% |
| PtrWRKY98 | Potri.018G139300.1.v3.0 | 58 | 19.33% | 14 | 4.67% | 228 | 76.00% |
| PtrWRKY99 | Potri.019G053900.1.v3.0 | 9 | 6.47% | 11 | 7.91% | 119 | 85.61% |
| PtrWRKY100 | Potri.019G059300.1.v3.0 | 20 | 7.97% | 19 | 7.57% | 212 | 84.46% |
| PtrWRKY101 | Potri.019G123500.1.v3.0 | 29 | 6.04% | 35 | 7.29% | 416 | 86.67% |
| PtrWRKY102 | Potri.T043800.1.v3.0 | 27 | 14.52% | 17 | 9.14% | 142 | 76.34% |
