# Supplementary material for: Enhanced resistance to Botryosphaeria dothidea through upregulation of the lignin biosynthesis regulator WRKY11 in poplar
Source: Front Plant Sci. 2026 Feb 26;17:1737207. doi: 10.3389/fpls.2026.1737207 (PMC12979517; doi:10.3389/fpls.2026.1737207)
Supplement: Supplementary file 5 [file Table5.pdf]

Table S5. Predition of the protein structure homology-modelling in PtrWRKYs

Name in this paper	Locus tag	Template	Sequence Identity	Description	Homology-modelling
PtrWRKY1	Potri.001G002400.1.v3.0	A0A4U5PP33.1.A	92.74%	WRKY transcription factor 44-like isoform X1	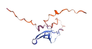
PtrWRKY2	Potri.001G044500.1.v3.0	A0A5P9K1S7.1.A	85.17%	Putative WRKY transcription factor	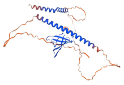
PtrWRKY3	Potri.001G058800.1.v3.0	A0A5P9NZM4.1.A	96.88%	WRKY75	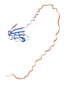
PtrWRKY4	Potri.001G092900.1.v3.0	A0A1U8JY19.1.A	71.47%	Probable WRKY transcription factor 41	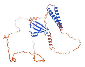
PtrWRKY5	Potri.001G099000.1.v3.0	A0A4U5PKN5.1.A	94.12%	WRKY domain-containing protein	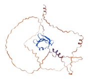
PtrWRKY6	Potri.001G121300.1.v3.0	A0A4U5P620.1.A	95.86%	WRKY transcription factor 22	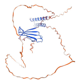
PtrWRKY7	Potri.001G208600.1.v3.0	A0A4U5NM25.1.A	94.91%	WRKY transcription factor 9 family protein	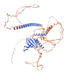
PtrWRKY8	Potri.001G328000.1.v3.0	A0A6N2MRY8.1.A	88.37%	WRKY domain-containing protein	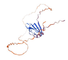
PtrWRKY9	Potri.001G352400.1.v3.0	A0A4V6XX87.1.A	97.76%	WRKY transcription factor 29	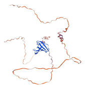
PtrWRKY10	Potri.001G361600.1.v3.0	A0A2I4KKT0.1.A	99.82%	WRKY family protein	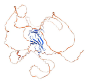
PtrWRKY11	Potri.001G460600.1.v3.0	A0A4U5N8Z7.1.A	99.05%	Putative WRKY transcription factor 14	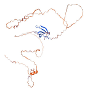
PtrWRKY12	Potri.001G472800.1.v3.0	A0A6A1VPZ4.1.A	74.14%	Putative WRKY transcription factor 2	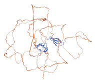
PtrWRKY13	Potri.002G043500.1.v3.0	A0A2P5FB72.1.A	82.76%	WRKY domain containing protein	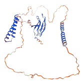
PtrWRKY14	Potri.002G059100.1.v3.0	A0A4U5Q1M6.1.A	87.27%	WRKY domain-containing protein	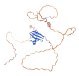

PtrWRKY15	Potri.002G123300.1.v3.0	C9DI03.1.A	96.36%	WRKY transcription factor 14	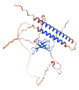
PtrWRKY16	Potri.002G138900.1.v3.0	I1JMP1.1.A	86.21%	WRKY domain-containing protein	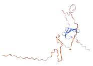
PtrWRKY17	Potri.002G164400.1.v3.0	A0A4V6ABW1.1.A	88.15%	WRKY transcription factor 23	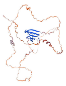
PtrWRKY18	Potri.002G164900.1.v3.0	B9GQD9.1.A	100.00%	WRKY domain-containing protein	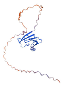
PtrWRKY19	Potri.002G168700.1.v3.0	A0A4U5QVE1.1.A	83.20%	WRKY domain-containing protein	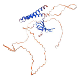
PtrWRKY20	Potri.002G186600.1.v3.0	B9GTA4.1.A	100.00%	WRKY domain-containing protein	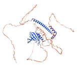
PtrWRKY21	Potri.002G193000.1.v3.0	A0A4U5LT41.1.A	98.11%	WRKY transcription factor	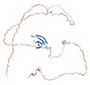
PtrWRKY22	Potri.002G195300.2.v3.0	A0A3S9LKC8.1.A	97.55%	WRKY DNA-binding protein 69	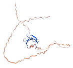
PtrWRKY23	Potri.002G221600.1.v3.0	A0A835JJN4.1.A	80.49%	WRKY domain-containing protein	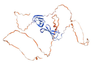
PtrWRKY24	Potri.002G228400.1.v3.0	A0A2K2BN03.1.A	100.00%	WRKY domain-containing protein	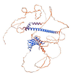
PtrWRKY25	Potri.003G111900.1.v3.0	A0A4U5PUL8.1.A	95.07%	Putative WRKY transcription factor 17	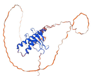
PtrWRKY26	Potri.003G132700.1.v3.0	A0A835N0R0.1.A	90.00%	WRKY domain-containing protein	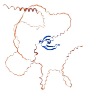
PtrWRKY27	Potri.003G138600.1.v3.0	B9GXG9.1.A	100.00%	WRKY domain-containing protein	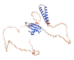
PtrWRKY28	Potri.003G169100.1.v3.0	A0A0R4J614.1.A	70.20%	WRKY transcription factor 53	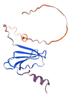
PtrWRKY29	Potri.003G182200.2.v3.0	S5CKF6.1.A	76.97%	WRKY transcription factor 27	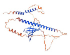
PtrWRKY30	Potri.004G007500.1.v3.0	A0A4U5Q2B9.1.A	84.83%	WRKY transcription factor 42 family protein	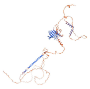
PtrWRKY31	Potri.004G060400.1.v3.0	A0A835JT08.1.A	87.79%	WRKY domain-containing protein	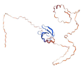

PtrWRKY32	Potri.004G060900.1.v3.0	A0A835JT08.1.A	87.79%	WRKY domain-containing protein	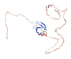
PtrWRKY33	Potri.004G072000.1.v3.0	B9H3N0.1.A	100.00%	WRKY domain-containing protein	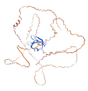
PtrWRKY34	Potri.004G120800.1.v3.0	A0A6M2EXS4.1.A	93.88%	WRKY domain-containing protein	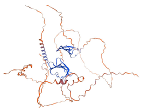
PtrWRKY35	Potri.005G055300.1.v3.0	A0A5B6ZNL3.1.A	77.34%	Putative WRKY transcription factor 74	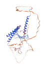
PtrWRKY36	Potri.005G085200.1.v3.0	A0A2K2ADP4.1.A	100.00%	WRKY domain-containing protein	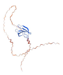
PtrWRKY37	Potri.005G086400.1.v3.0	A0A2K2ADS2.1.A	100.00%	WRKY domain-containing protein	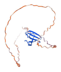
PtrWRKY38	Potri.005G141400.1.v3.0	A0A0R0FEJ5.1.A	70.13%	WRKY domain-containing protein	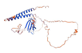
PtrWRKY39	Potri.005G203200.1.v3.0	A0A4U5Q1M6.1.A	99.38%	WRKY domain-containing protein	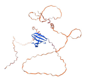
PtrWRKY40	Potri.005G219500.1.v3.0	W9RBZ3.1.A	85.80%	Putative WRKY transcription factor 21	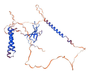
PtrWRKY41	Potri.006G072400.1.v3.0	C6TKE5.1.A	72.08%	WRKY transcription factor 33	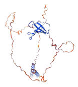
PtrWRKY42	Potri.006G087000.1.v3.0	G1ESZ7.1.A	91.12%	WRKY32	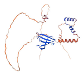
PtrWRKY43	Potri.006G105300.1.v3.0	A0A2K2A022.1.A	100.00%	WRKY domain-containing protein	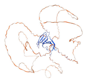
PtrWRKY44	Potri.006G109100.1.v3.0	A0A6M2EIX9.1.A	92.17%	WRKY domain-containing protein	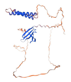
PtrWRKY45	Potri.006G133200.8.v3.0	A0A5B7BFW8.1.A	70.61%	Putative WRKY transcription factor 44	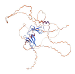
PtrWRKY46	Potri.006G184800.1.v3.0	A0A6A6MEI8.1.A	72.87%	WRKY domain-containing protein	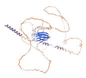
PtrWRKY47	Potri.006G224100.1.v3.0	B9HDB0.1.A	100.00%	WRKY domain-containing protein	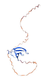
PtrWRKY48	Potri.006G263600.1.v3.0	A0A835J746.1.A	77.64%	WRKY domain-containing protein	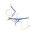

PtrWRKY49	Potri.006G264000.1.v3.0	A0A2K2A8P9.1.A	100.00%	WRKY domain-containing protein	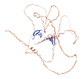
PtrWRKY50	Potri.007G047400.1.v3.0	B0LUS5.1.A	72.81%	WRKY15a	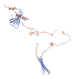
PtrWRKY51	Potri.007G078200.1.v3.0	A0A6N2ME35.1.A	87.50%	WRKY domain-containing protein	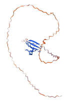
PtrWRKY52	Potri.007G079800.1.v3.0	A0A2K1ZR01.1.A	100.00%	WRKY domain-containing protein	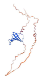
PtrWRKY53	Potri.008G091900.1.v3.0	A0A5N5L6W2.1.A	82.24%	WRKY domain-containing protein	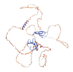
PtrWRKY54	Potri.008G094000.1.v3.0	I1JEC8.1.A	70.33%	WRKY domain-containing protein	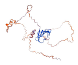
PtrWRKY55	Potri.008G103300.1.v3.0	B9HIL9.1.A	100.00%	WRKY domain-containing protein	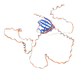
PtrWRKY56	Potri.010G147700.1.v3.0	B9HX42.1.A	100.00%	WRKY domain-containing protein	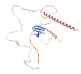
PtrWRKY57	Potri.010G160100.2.v3.0	A0A4U5MWP9.1.A	98.29%	WRKY domain-containing protein	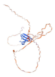
PtrWRKY58	Potri.010G163000.1.v3.0	A0A5N5L6W2.1.A	89.43%	WRKY domain-containing protein	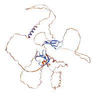
PtrWRKY59	Potri.011G007800.1.v3.0	A0A4U5Q2B9.1.A	97.64%	WRKY transcription factor 42 family protein	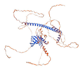
PtrWRKY60	Potri.011G070100.1.v3.0	A0A3Q9EDY4.1.A	96.64%	WRKY transcription factor 65	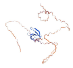
PtrWRKY61	Potri.011G079300.1.v3.0	A0A4V6XX87.1.A	84.59%	WRKY transcription factor 29	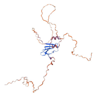
PtrWRKY62	Potri.011G087900.1.v3.0	A0A2C9W7B0.1.A	75.86%	WRKY domain-containing protein	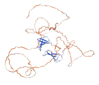
PtrWRKY63	Potri.011G157100.1.v3.0	B9I0X2.1.A	100.00%	WRKY domain-containing protein	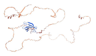
PtrWRKY64	Potri.011G169300.1.v3.0	U5G0P4.1.A	100.00%	WRKY domain-containing protein	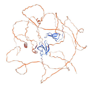
PtrWRKY65	Potri.012G031700.1.v3.0	A0A6N2K9N2.1.A	86.79%	WRKY domain-containing protein	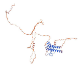
PtrWRKY66	Potri.012G101000.1.v3.0	B9I3U6.1.A	100.00%	WRKY domain-containing protein	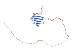

PtrWRKY67	Potri.013G042600.1.v3.0	A0A5B6ZNL3.1.A	75.71%	Putative WRKY transcription factor 74	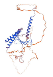
PtrWRKY68	Potri.013G086000.1.v3.0	A0A2K1Y335.1.A	100.00%	WRKY domain-containing protein	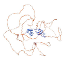
PtrWRKY69	Potri.013G090300.1.v3.0	A0A2K1Y395.1.A	100.00%	WRKY domain-containing protein	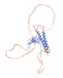
PtrWRKY70	Potri.013G090400.1.v3.0	A0A4U5PL96.1.A	95.17%	WRKY transcription factor 55-like	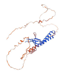
PtrWRKY71	Potri.013G153400.1.v3.0	I1MBH2.1.A	74.60%	WRKY transcription factor 49	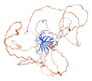
PtrWRKY72	Potri.014G009500.1.v3.0	B9NDT5.1.A	100.00%	WRKY domain-containing protein	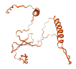
PtrWRKY73	Potri.014G024200.1.v3.0	A0A835JK23.1.A	94.41%	WRKY domain-containing protein	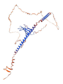
PtrWRKY74	Potri.014G050000.1.v3.0	A0A836XX10.1.A	94.25%	UniProtKB entry unknown, most likely obsolete	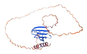
PtrWRKY75	Potri.014G090300.1.v3.0	A0A4V6ABW1.1.A	96.85%	WRKY transcription factor 23	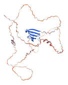
PtrWRKY76	Potri.014G090700.1.v3.0	A0A5D2PG25.1.A	75.40%	WRKY domain-containing protein	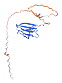
PtrWRKY77	Potri.014G096200.1.v3.0	A0A4U5QVE1.1.A	95.62%	WRKY domain-containing protein	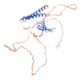
PtrWRKY78	Potri.014G111900.1.v3.0	A0A835MKK7.1.A	91.43%	WRKY domain-containing protein	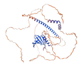
PtrWRKY79	Potri.014G118200.1.v3.0	A0A4U5QUF4.1.A	96.55%	WRKY transcription factor	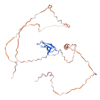
PtrWRKY80	Potri.014G119800.1.v3.0	A0A836AVL0.1.A	88.17%	UniProtKB entry unknown, most likely obsolete	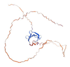
PtrWRKY81	Potri.014G155100.1.v3.0	A0A2K1XWC2.1.A	100.00%	WRKY domain-containing protein	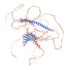
PtrWRKY82	Potri.014G164300.1.v3.0	U5FVL1.1.A	100.0%%	WRKY domain-containing protein	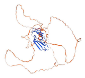
PtrWRKY83	Potri.015G064100.1.v3.0	A0A2K1XJ02.1.A	100.00%	WRKY domain-containing protein	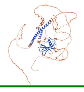

PtrWRKY84	Potri.015G099200.1.v3.0	A0A4U5Q921.1.A	98.88%	WRKY domain-containing protein	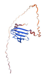
PtrWRKY85	Potri.016G083600.5.v3.0	A0A2P2LMK3.1.A	72.67%	Uncharacterized protein	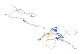
PtrWRKY86	Potri.016G099900.1.v3.0	G1ESZ7.1.A	77.05%	WRKY32	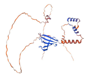
PtrWRKY87	Potri.016G128300.1.v3.0	A0A4U5P5X8.1.A	97.58%	WRKY transcription factor 17	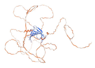
PtrWRKY88	Potri.016G137900.1.v3.0	A0A2K1XFB7.1.A	100.00%	WRKY family protein	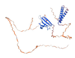
PtrWRKY89	Potri.017G079500.1.v3.0	A0A4U5PPL2.1.A	97.40%	WRKY transcription factor 72 family protein	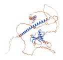
PtrWRKY90	Potri.017G088300.1.v3.0	A0A6M2EXS4.1.A	89.58%	WRKY domain-containing protein	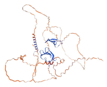
PtrWRKY91	Potri.017G104800.1.v3.0	A0A060AFA1.1.A	100.00%	WRKY66	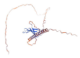
PtrWRKY92	Potri.017G149000.1.v3.0	B9MX64.1.A	100.00%	WRKY domain-containing protein	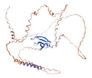
PtrWRKY93	Potri.018G008500.1.v3.0	C9DI13.1.A	97.63%	WRKY transcription factor 24	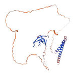
PtrWRKY94	Potri.018G019000.1.v3.0	A0A6N2NB63.1.A	71.43%	WRKY domain-containing protein	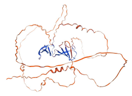
PtrWRKY95	Potri.018G019700.1.v3.0	A0A835J4V1.1.A	81.92%	WRKY domain-containing protein	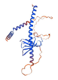
PtrWRKY96	Potri.018G019800.1.v3.0	A0A835J746.1.A	86.67%	WRKY domain-containing protein	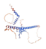
PtrWRKY97	Potri.018G107000.1.v3.0	A0A6A6MEI8.1.A	72.80%	WRKY domain-containing protein	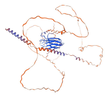
PtrWRKY98	Potri.018G139300.1.v3.0	C6TKE5.1.A	71.43%	WRKY transcription factor 33	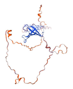
PtrWRKY99	Potri.019G053900.1.v3.0	G8GB84.1.A	93.02%	WRKY transcription factor	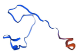
PtrWRKY100	Potri.019G059300.1.v3.0	A0A2K1WQC3.1.A	100.00%	WRKY domain-containing protein	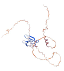

PtrWRKY101	Potri.019G123500.1.v3.0	I1MBH2.1.A	75.39%	WRKY transcription factor 49	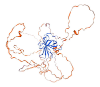
PtrWRKY102	Potri.T043800.1.v3.0	A0A4U5QD46.1.A	83.24%	Putative WRKY transcription factor 75	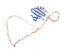
