# Supplementary material for: Enhanced resistance to Botryosphaeria dothidea through upregulation of the lignin biosynthesis regulator WRKY11 in poplar
Source: Front Plant Sci. 2026 Feb 26;17:1737207. doi: 10.3389/fpls.2026.1737207 (PMC12979517; doi:10.3389/fpls.2026.1737207)
Supplement: Supplementary file 7 [file Table7.doc]

**Table S7**. Subcellular localization and gene coordinate of PtrWRKYs proteins

| Name in this paper | Locus tag | Subcellular localization | Chromosome |
| --- | --- | --- | --- |
| PtrWRKY1 | Potri.001G002400.1.v3.0 | Nucleus | Chromosome 1 |
| PtrWRKY2 | Potri.001G044500.1.v3.0 | Nucleus | Chromosome 1 |
| PtrWRKY3 | Potri.001G058800.1.v3.0 | Nucleus | Chromosome 1 |
| PtrWRKY4 | Potri.001G092900.1.v3.0 | Nucleus | Chromosome 1 |
| PtrWRKY5 | Potri.001G099000.1.v3.0 | Nucleus | Chromosome 1 |
| PtrWRKY6 | Potri.001G121300.1.v3.0 | Nucleus | Chromosome 1 |
| PtrWRKY7 | Potri.001G208600.1.v3.0 | Nucleus | Chromosome 1 |
| PtrWRKY8 | Potri.001G328000.1.v3.0 | Nucleus | Chromosome 1 |
| PtrWRKY9 | Potri.001G352400.1.v3.0 | Nucleus | Chromosome 1 |
| PtrWRKY10 | Potri.001G361600.1.v3.0 | Nucleus | Chromosome 1 |
| PtrWRKY11 | Potri.001G460600.1.v3.0 | Nucleus | Chromosome 1 |
| PtrWRKY12 | Potri.001G472800.1.v3.0 | Nucleus | Chromosome 1 |
| PtrWRKY13 | Potri.002G043500.1.v3.0 | Nucleus | Chromosome 2 |
| PtrWRKY14 | Potri.002G059100.1.v3.0 | Nucleus | Chromosome 2 |
| PtrWRKY15 | Potri.002G123300.1.v3.0 | Nucleus | Chromosome 2 |
| PtrWRKY16 | Potri.002G138900.1.v3.0 | Nucleus | Chromosome 2 |
| PtrWRKY17 | Potri.002G164400.1.v3.0 | Nucleus | Chromosome 2 |
| PtrWRKY18 | Potri.002G164900.1.v3.0 | Peroxisome | Chromosome 2 |
| PtrWRKY19 | Potri.002G168700.1.v3.0 | Nucleus | Chromosome 2 |
| PtrWRKY20 | Potri.002G186600.1.v3.0 | Nucleus | Chromosome 2 |
| PtrWRKY21 | Potri.002G193000.1.v3.0 | Nucleus | Chromosome 2 |
| PtrWRKY22 | Potri.002G195300.2.v3.0 | Nucleus | Chromosome 2 |
| PtrWRKY23 | Potri.002G221600.1.v3.0 | Nucleus | Chromosome 2 |
| PtrWRKY24 | Potri.002G228400.1.v3.0 | Nucleus | Chromosome 2 |
| PtrWRKY25 | Potri.003G111900.1.v3.0 | Nucleus | Chromosome 3 |
| PtrWRKY26 | Potri.003G132700.1.v3.0 | Nucleus | Chromosome 3 |
| PtrWRKY27 | Potri.003G138600.1.v3.0 | Nucleus | Chromosome 3 |
| PtrWRKY28 | Potri.003G169100.1.v3.0 | Nucleus | Chromosome 3 |
| PtrWRKY29 | Potri.003G182200.2.v3.0 | Nucleus | Chromosome 3 |
| PtrWRKY30 | Potri.004G007500.1.v3.0 | Nucleus | Chromosome 4 |
| PtrWRKY31 | Potri.004G060400.1.v3.0 | Nucleus | Chromosome 4 |
| PtrWRKY32 | Potri.004G060900.1.v3.0 | Nucleus | Chromosome 4 |
| PtrWRKY33 | Potri.004G072000.1.v3.0 | Nucleus | Chromosome 4 |
| PtrWRKY34 | Potri.004G120800.1.v3.0 | Nucleus | Chromosome 4 |
| PtrWRKY35 | Potri.005G055300.1.v3.0 | Nucleus | Chromosome 5 |
| PtrWRKY36 | Potri.005G085200.1.v3.0 | Nucleus | Chromosome 5 |
| PtrWRKY37 | Potri.005G086400.1.v3.0 | Nucleus | Chromosome 5 |
| PtrWRKY38 | Potri.005G141400.1.v3.0 | Nucleus | Chromosome 5 |
| PtrWRKY39 | Potri.005G203200.1.v3.0 | Nucleus | Chromosome 5 |
| PtrWRKY40 | Potri.005G219500.1.v3.0 | Nucleus | Chromosome 5 |
| PtrWRKY41 | Potri.006G072400.1.v3.0 | Nucleus | Chromosome 6 |
| PtrWRKY42 | Potri.006G087000.1.v3.0 | Nucleus | Chromosome 6 |
| PtrWRKY43 | Potri.006G105300.1.v3.0 | Nucleus | Chromosome 6 |
| PtrWRKY44 | Potri.006G109100.1.v3.0 | Nucleus | Chromosome 6 |
| PtrWRKY45 | Potri.006G133200.8.v3.0 | Nucleus | Chromosome 6 |
| PtrWRKY46 | Potri.006G184800.1.v3.0 | Nucleus | Chromosome 6 |
| PtrWRKY47 | Potri.006G224100.1.v3.0 | Nucleus | Chromosome 6 |
| PtrWRKY48 | Potri.006G263600.1.v3.0 | Nucleus | Chromosome 6 |
| PtrWRKY49 | Potri.006G264000.1.v3.0 | Nucleus | Chromosome 6 |
| PtrWRKY50 | Potri.007G047400.1.v3.0 | Nucleus | Chromosome 7 |
| PtrWRKY51 | Potri.007G078200.1.v3.0 | Nucleus | Chromosome 7 |
| PtrWRKY52 | Potri.007G079800.1.v3.0 | Nucleus | Chromosome 7 |
| PtrWRKY53 | Potri.008G091900.1.v3.0 | Nucleus | Chromosome 8 |
| PtrWRKY54 | Potri.008G094000.1.v3.0 | Nucleus | Chromosome 8 |
| PtrWRKY55 | Potri.008G103300.1.v3.0 | Nucleus | Chromosome 8 |
| PtrWRKY56 | Potri.010G147700.1.v3.0 | Nucleus | Chromosome 10 |
| PtrWRKY57 | Potri.010G160100.2.v3.0 | Nucleus | Chromosome 10 |
| PtrWRKY58 | Potri.010G163000.1.v3.0 | Nucleus | Chromosome 10 |
| PtrWRKY59 | Potri.011G007800.1.v3.0 | Nucleus | Chromosome 11 |
| PtrWRKY60 | Potri.011G070100.1.v3.0 | Nucleus | Chromosome 11 |
| PtrWRKY61 | Potri.011G079300.1.v3.0 | Nucleus | Chromosome 11 |
| PtrWRKY62 | Potri.011G087900.1.v3.0 | Nucleus | Chromosome 11 |
| PtrWRKY63 | Potri.011G157100.1.v3.0 | Nucleus | Chromosome 11 |
| PtrWRKY64 | Potri.011G169300.1.v3.0 | Nucleus | Chromosome 11 |
| PtrWRKY65 | Potri.012G031700.1.v3.0 | Nucleus | Chromosome 12 |
| PtrWRKY66 | Potri.012G101000.1.v3.0 | Nucleus | Chromosome 12 |
| PtrWRKY67 | Potri.013G042600.1.v3.0 | Nucleus | Chromosome 13 |
| PtrWRKY68 | Potri.013G086000.1.v3.0 | Nucleus | Chromosome 13 |
| PtrWRKY69 | Potri.013G090300.1.v3.0 | Nucleus | Chromosome 13 |
| PtrWRKY70 | Potri.013G090400.1.v3.0 | Nucleus | Chromosome 13 |
| PtrWRKY71 | Potri.013G153400.1.v3.0 | Nucleus | Chromosome 13 |
| PtrWRKY72 | Potri.014G009500.1.v3.0 | Nucleus | Chromosome 14 |
| PtrWRKY73 | Potri.014G024200.1.v3.0 | Nucleus | Chromosome 14 |
| PtrWRKY74 | Potri.014G050000.1.v3.0 | Nucleus | Chromosome 14 |
| PtrWRKY75 | Potri.014G090300.1.v3.0 | Nucleus | Chromosome 14 |
| PtrWRKY76 | Potri.014G090700.1.v3.0 | Peroxisome | Chromosome 14 |
| PtrWRKY77 | Potri.014G096200.1.v3.0 | Nucleus | Chromosome 14 |
| PtrWRKY78 | Potri.014G111900.1.v3.0 | Nucleus | Chromosome 14 |
| PtrWRKY79 | Potri.014G118200.1.v3.0 | Nucleus | Chromosome 14 |
| PtrWRKY80 | Potri.014G119800.1.v3.0 | Nucleus | Chromosome 14 |
| PtrWRKY81 | Potri.014G155100.1.v3.0 | Nucleus | Chromosome 14 |
| PtrWRKY82 | Potri.014G164300.1.v3.0 | Nucleus | Chromosome 14 |
| PtrWRKY83 | Potri.015G064100.1.v3.0 | Nucleus | Chromosome 15 |
| PtrWRKY84 | Potri.015G099200.1.v3.0 | Nucleus | Chromosome 15 |
| PtrWRKY85 | Potri.016G083600.5.v3.0 | Nucleus | Chromosome 16 |
| PtrWRKY86 | Potri.016G099900.1.v3.0 | Nucleus | Chromosome 16 |
| PtrWRKY87 | Potri.016G128300.1.v3.0 | Nucleus | Chromosome 16 |
| PtrWRKY88 | Potri.016G137900.1.v3.0 | Nucleus | Chromosome 16 |
| PtrWRKY89 | Potri.017G079500.1.v3.0 | Nucleus | Chromosome 17 |
| PtrWRKY90 | Potri.017G088300.1.v3.0 | Chloroplast | Chromosome 17 |
| PtrWRKY91 | Potri.017G104800.1.v3.0 | Nucleus | Chromosome 17 |
| PtrWRKY92 | Potri.017G149000.1.v3.0 | Nucleus | Chromosome 17 |
| PtrWRKY93 | Potri.018G008500.1.v3.0 | Nucleus | Chromosome 18 |
| PtrWRKY94 | Potri.018G019000.1.v3.0 | Nucleus | Chromosome 18 |
| PtrWRKY95 | Potri.018G019700.1.v3.0 | Nucleus | Chromosome 18 |
| PtrWRKY96 | Potri.018G019800.1.v3.0 | Nucleus | Chromosome 18 |
| PtrWRKY97 | Potri.018G107000.1.v3.0 | Nucleus | Chromosome 18 |
| PtrWRKY98 | Potri.018G139300.1.v3.0 | Nucleus | Chromosome 18 |
| PtrWRKY99 | Potri.019G053900.1.v3.0 | Cytoplasm | Chromosome 19 |
| PtrWRKY100 | Potri.019G059300.1.v3.0 | Nucleus | Chromosome 19 |
| PtrWRKY101 | Potri.019G123500.1.v3.0 | Nucleus | Chromosome 19 |
| PtrWRKY102 | Potri.T043800.1.v3.0 | Nucleus | Scaffold 41 |
