# Supplementary material for: Enhanced resistance to Botryosphaeria dothidea through upregulation of the lignin biosynthesis regulator WRKY11 in poplar
Source: Front Plant Sci. 2026 Feb 26;17:1737207. doi: 10.3389/fpls.2026.1737207 (PMC12979517; doi:10.3389/fpls.2026.1737207)
Supplement: Supplementary file 8 [file Table8.doc]

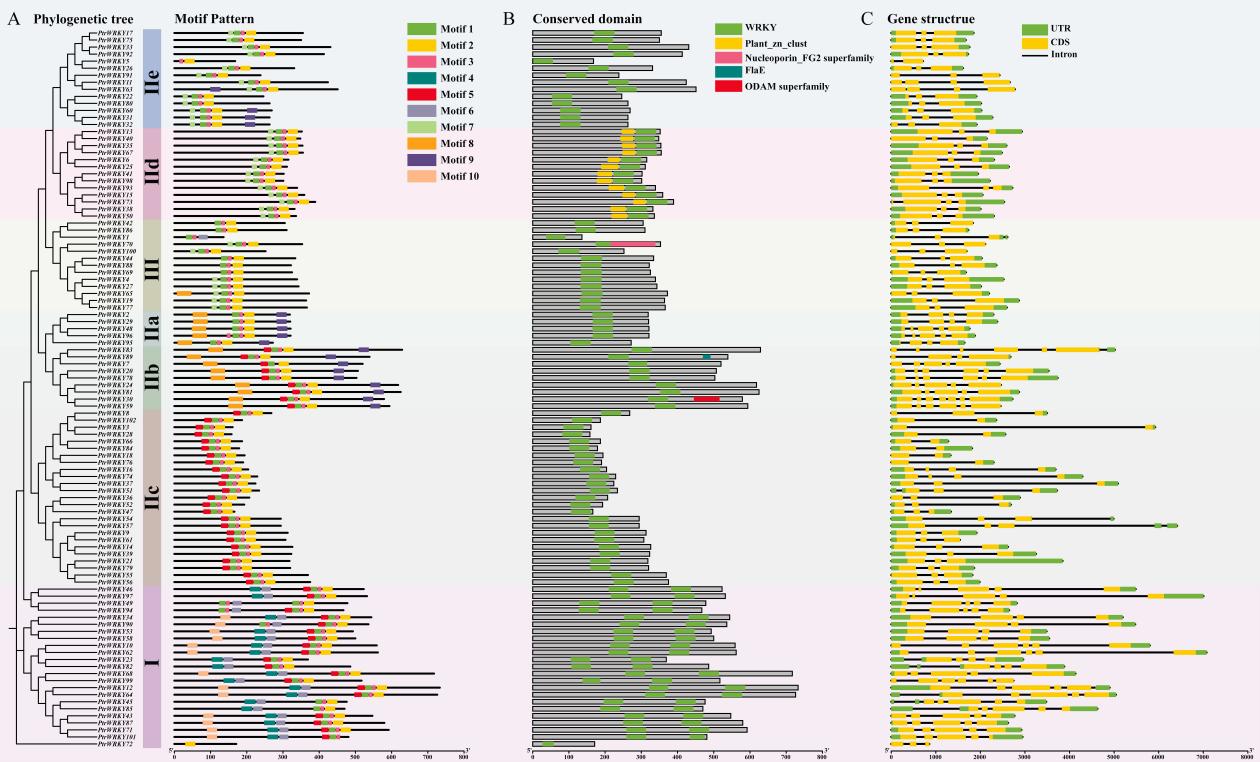


Fig. S1 Genomic structural characterization of 102 PtrWRKYs. A: Motif pattern analysis. B: Conserved domain analysis. C: Gene structure analysis. Yellow boxes represent coding sequences (CDS); green boxes indicate untranslated regions (UTRs); black lines denote intron.


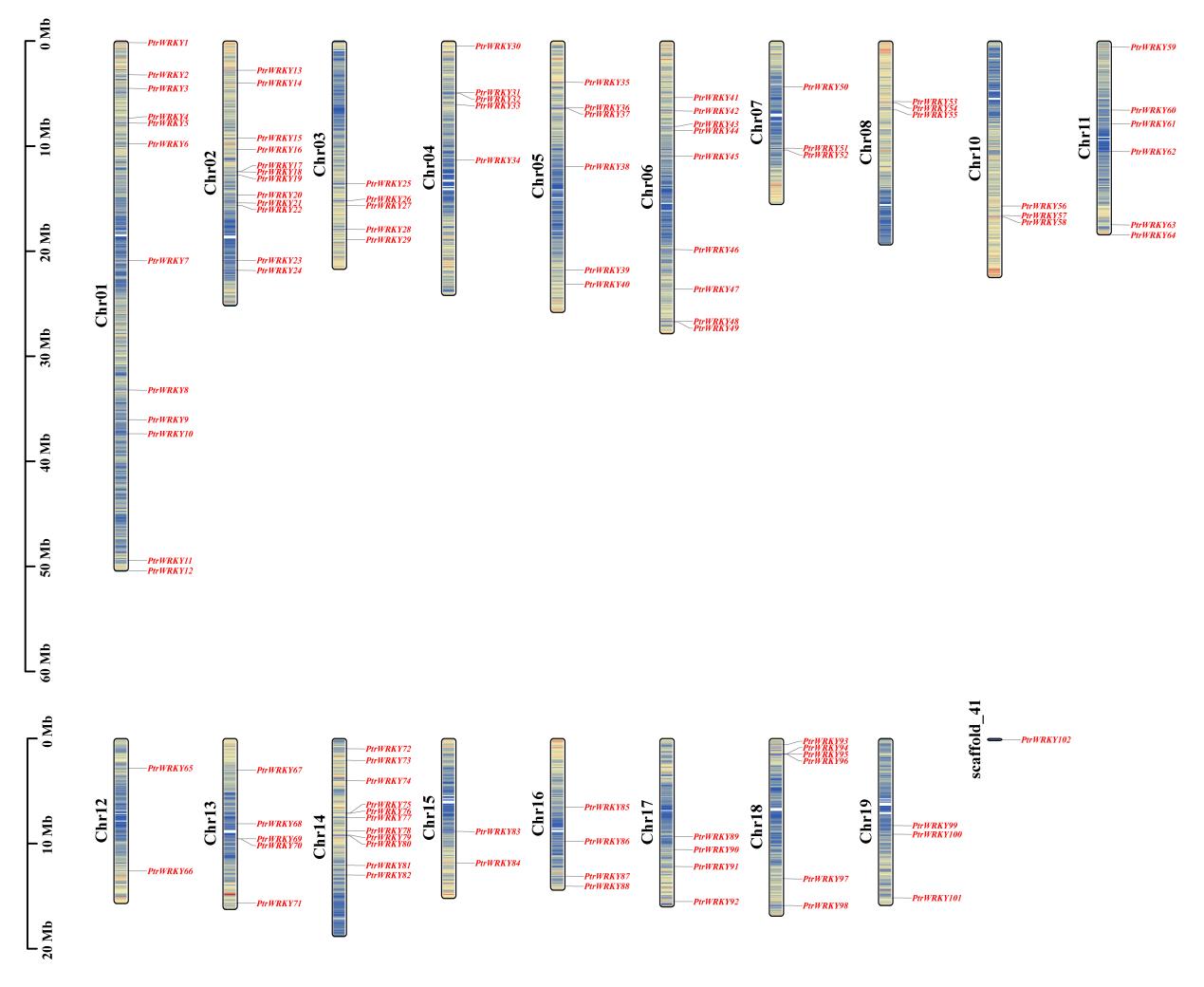


Fig. S2 Chromosomal distribution of 102 PtrWRKYs in *P. trichocarpa*, visualized using TBtools. Chr1–Chr19 are labeled numerically, while Scaffold 41 is annotated as an anchored genomic region.


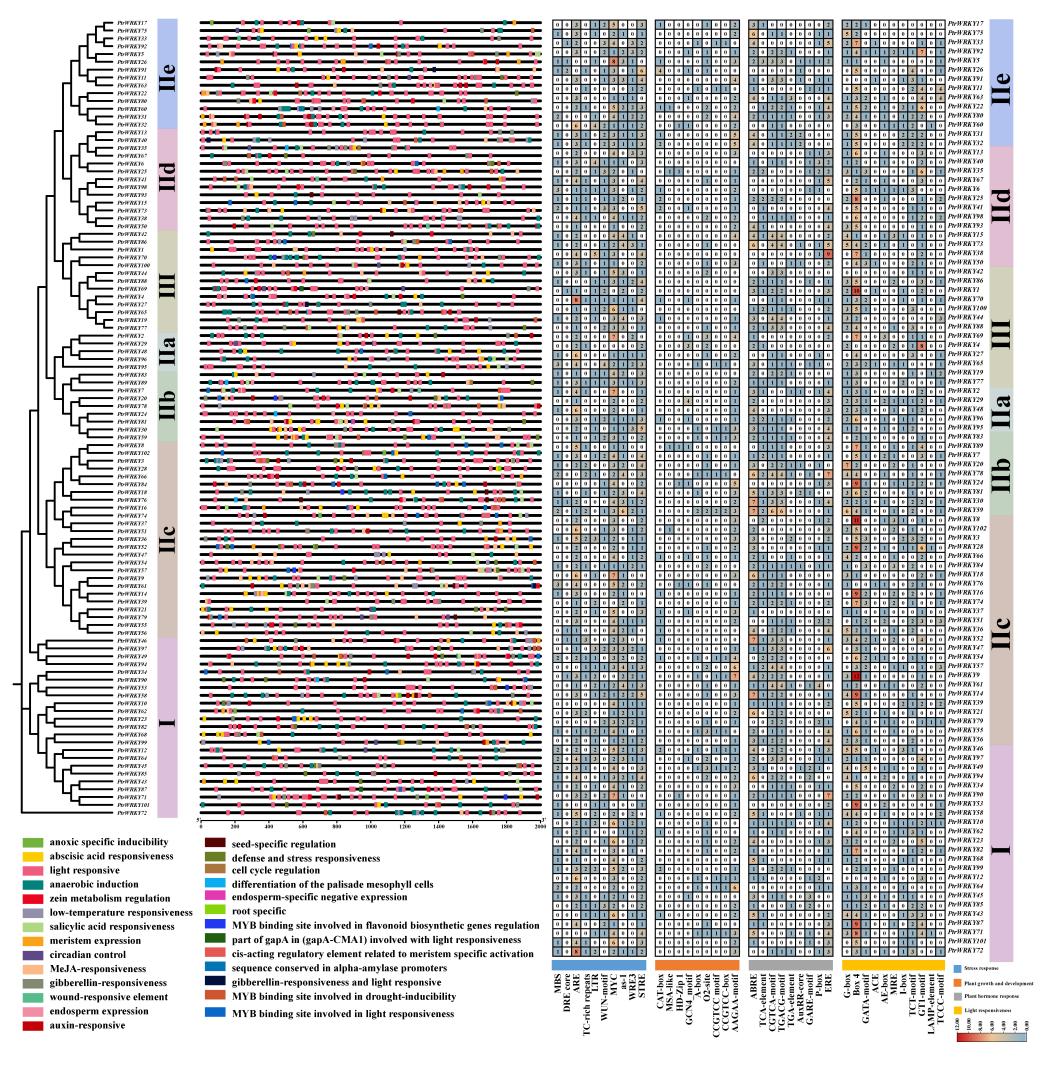


Fig. S3 Analysis of cis-acting elements in the promoter regions of 102 PtrWRKYs. Different colors indicate distinct types of cis-acting elements. Colored boxes represent specific heatmap modules. The color gradient in the heatmap reflects the relative abundance of cis-acting elements per gene. red color lump represents high expression quantity; blue color lump means a low expression quantity.


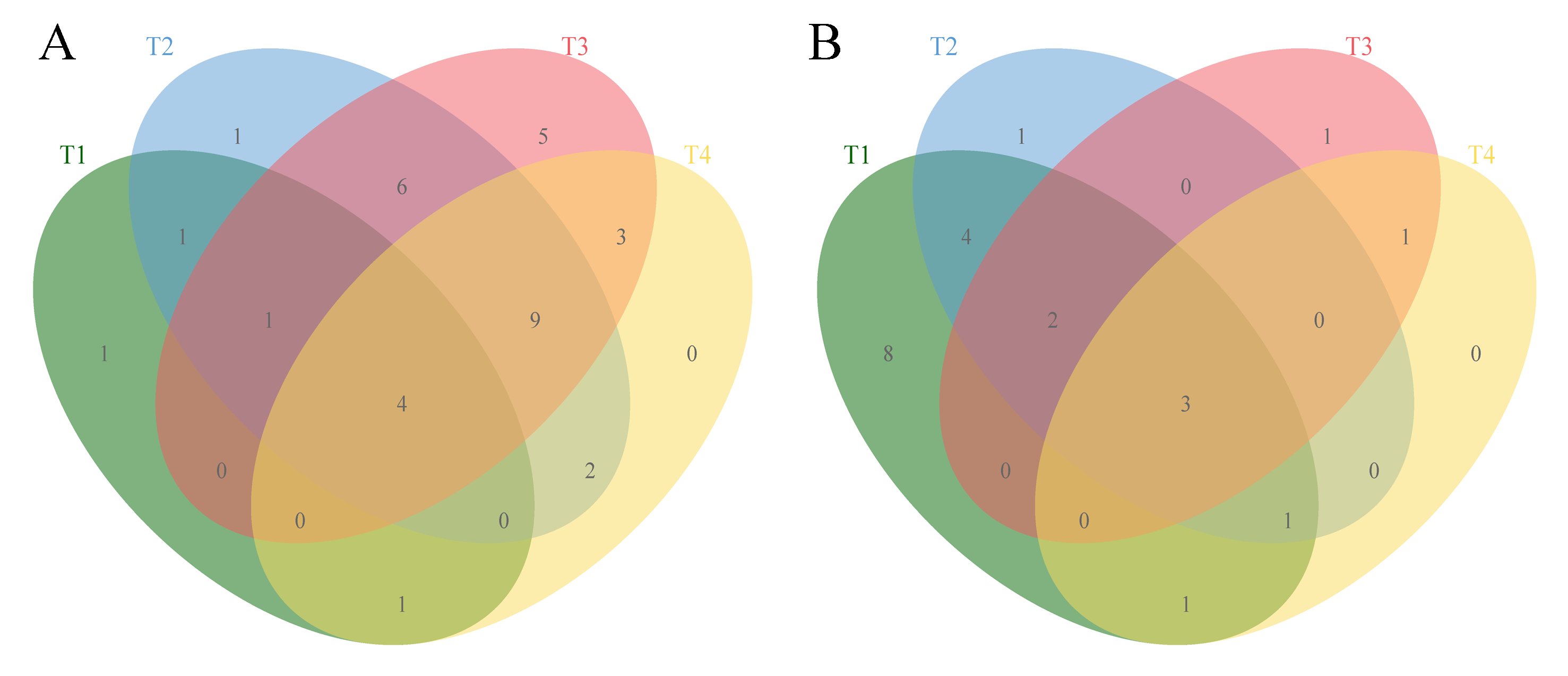


Fig. S4 Venn diagrams of DEG expression under distinct *B. dothidea* treatments. A: Up-regulated DEGs; B: Down-regulated DEGs.


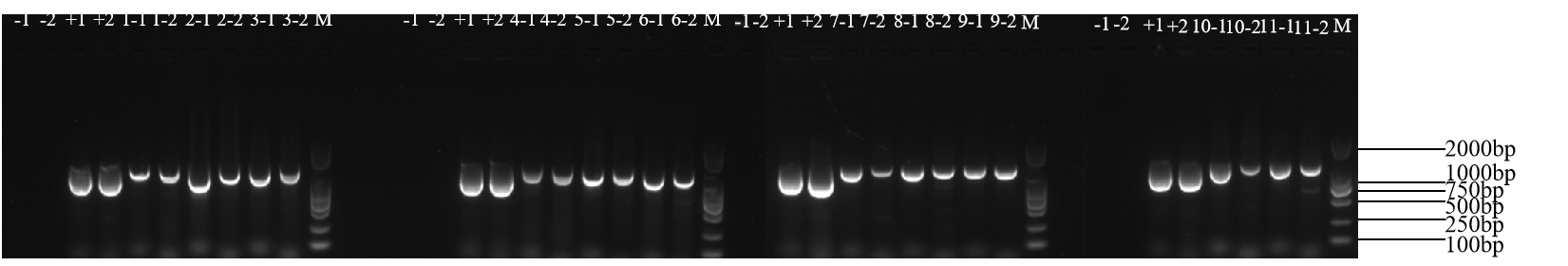


Fig. S5 Molecular detection in overexpressing putative transformant Pdpap of *PdpapWRKY11*. M: DL 2000 Marker; 1–11: Eleven overexpressing putative transformant lines were used as amplification templates;+: Positive control using PBI121-*PdpapWRKY11* plasmid as template; −: Negative control with water as template; −1: PCR detection of putative transformant lines using PBI121-F and*PdpapWRKY11*-R as primers; −2: PCR detection of putative transformant lines with *PdpapWRKY11*-F and PBI121-R as primers.


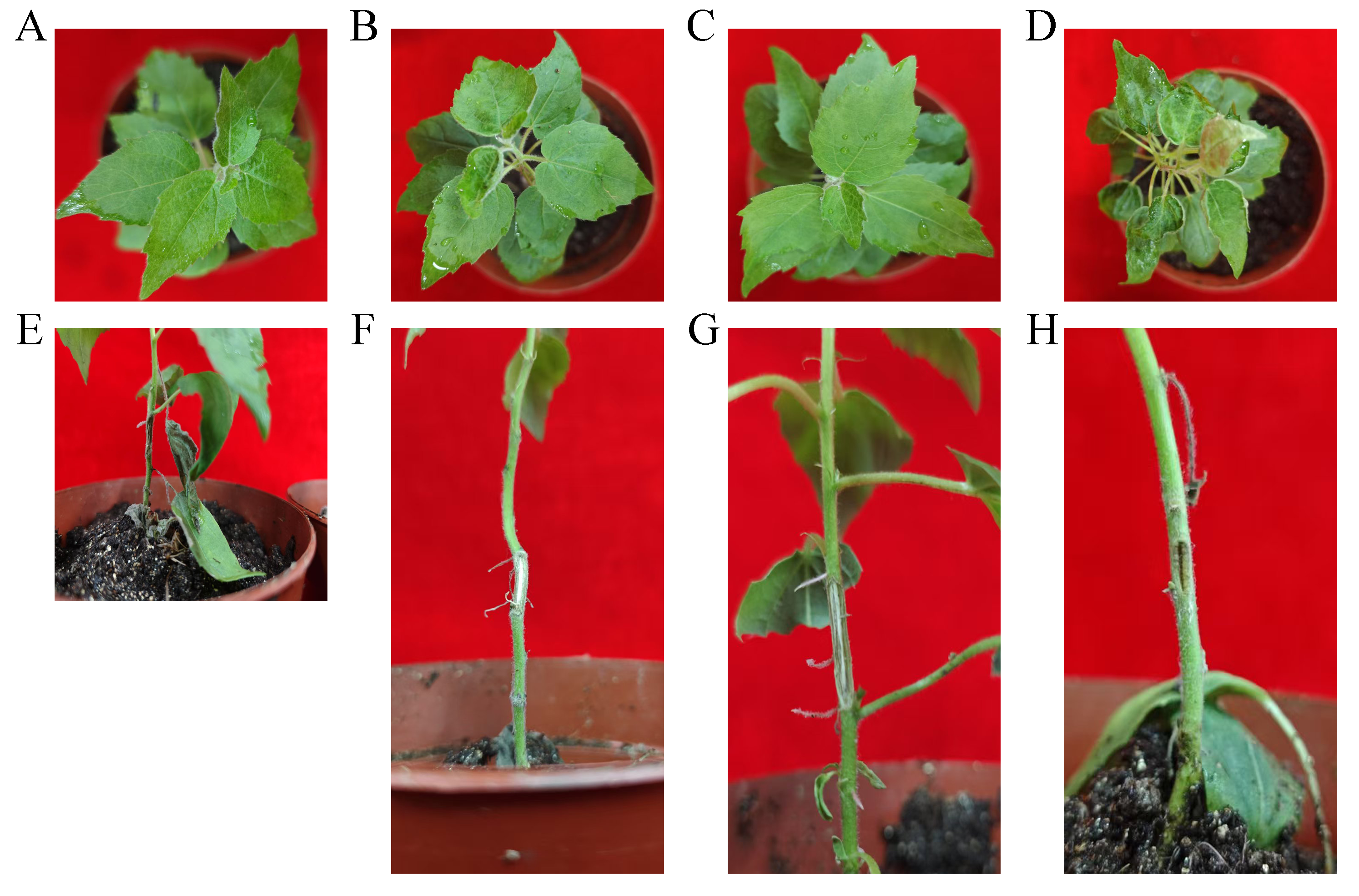


Fig. S6 Pathological manifestations. (A-D): Top view of growth status in WT Pdpap and OE4, OE7, OE10 at 20 d post-inoculation with *B. dothidea*; (E-H): Wound morphology in WT Pdpap and OE4, OE7, OE10 at 20 d post-inoculation with *B. dothidea*.


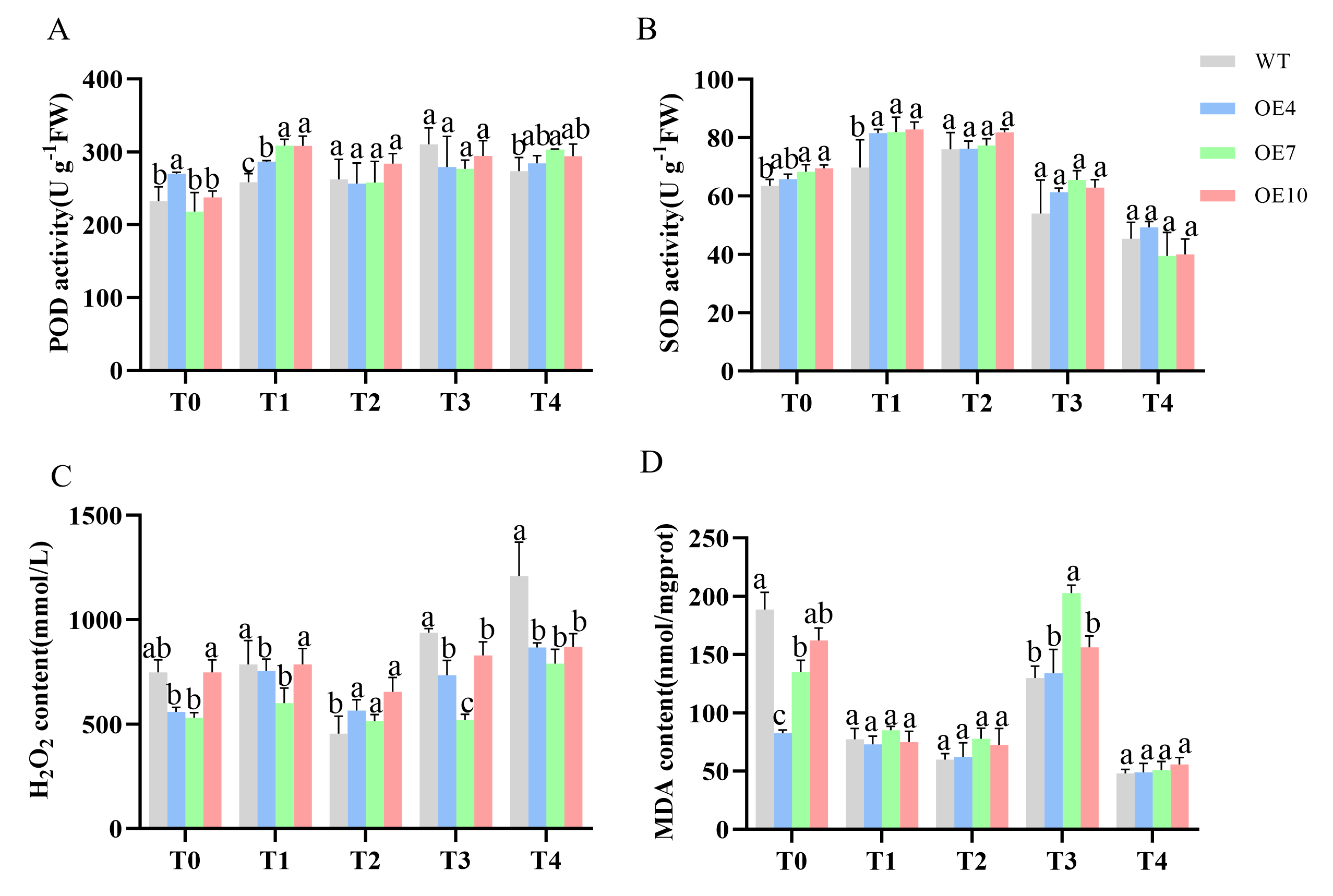


Fig. S7 Physiological characteristic analysis of wild Pdpap and *PdpapWRKY11* overexpression transformants infected with *B. dothidea*. A: Result of POD activity measurement; B: Result of SOD activity measurement; C: Result of H_2_O_2_ content measurement; D: Result of MDA content. T0−T4: Infection times were 0, 6, 12, 24, 48 h. Error bars represent standard deviation of three independent replicates. Significant differences (*P*<0.05) are indicated by different lowercase letters.
